# Pathogenic and likely pathogenic variant prevalence among the first 10,000 patients referred for next-generation cancer panel testing

**DOI:** 10.1038/gim.2015.166

**Published:** 2015-12-17

**Authors:** Lisa R. Susswein, Megan L. Marshall, Rachel Nusbaum, Kristen J. Vogel Postula, Scott M. Weissman, Lauren Yackowski, Erica M. Vaccari, Jeffrey Bissonnette, Jessica K. Booker, M. Laura Cremona, Federica Gibellini, Patricia D. Murphy, Daniel E. Pineda-Alvarez, Guido D. Pollevick, Zhixiong Xu, Gabi Richard, Sherri Bale, Rachel T. Klein, Kathleen S. Hruska, Wendy K. Chung

**Affiliations:** 1 GeneDx, Gaithersburg, Maryland, USA; 2 BioReference Laboratories, Elmwood Park, New Jersey, USA; 3 Department of Pediatrics and Medicine, Columbia University Medical Center, New York, New York, USA

**Keywords:** *BRCA1/2*, hereditary breast and ovarian cancer, hereditary cancer panels, Lynch syndrome, next generation sequencing

## Abstract

**Purpose:**

Germ-line testing for panels of cancer genes using next-generation sequencing is becoming more common in clinical care. We report our experience as a clinical laboratory testing both well-established, high-risk cancer genes (e.g., *BRCA1*/*2*, *MLH1*, *MSH2*) as well as more recently identified cancer genes (e.g., *PALB2*, *BRIP1*), many of which have increased but less well-defined penetrance.

*Genet Med* **18** 8, 823–832.

**Methods:**

Clinical genetic testing was performed on over 10,000 consecutive cases referred for evaluation of germ-line cancer genes, and results were analyzed for frequency of pathogenic or likely pathogenic variants, and were stratified by testing panel, gene, and clinical history.

*Genet Med* **18** 8, 823–832.

**Results:**

Overall, a molecular diagnosis was made in 9.0% of patients tested, with the highest yield in the Lynch syndrome/colorectal cancer panel. In patients with breast, ovarian, or colon/stomach cancer, positive yields were 9.7, 13.4, and 14.8%, respectively. Approximately half of the pathogenic variants identified in patients with breast or ovarian cancer were in genes other than *BRCA1*/*2*.

*Genet Med* **18** 8, 823–832.

**Conclusion:**

The high frequency of positive results in a wide range of cancer genes, including those of high penetrance and with clinical care guidelines, underscores both the genetic heterogeneity of hereditary cancer and the usefulness of multigene panels over genetic tests of one or two genes.

*Genet Med* **18** 8, 823–832.

## Introduction

Next-generation sequencing (NGS) has reduced the cost of sequencing, increased the capacity to analyze many genes in parallel, and enabled significant progress in our understanding of the genetic etiology and architecture of human disease. While hereditary breast and ovarian cancer has been a common indication for germ-line genetic evaluation since *BRCA1* and *BRCA2* were identified, many additional genes have subsequently been implicated in hereditary cancer.

Commercial laboratories began offering clinical NGS panels for inherited cancer within the past 3 years. Large panels can increase the yield of genetic testing for diseases with genetic heterogeneity or variable and overlapping phenotypes, and they have been used routinely for other indications, including hereditary cardiac and neurological diseases. One drawback to testing a larger number of genes, especially those that are less well characterized, is the potential for higher numbers of variants of uncertain clinical significance (VUSs).^1,2^ For many patients and providers, however, the possibility of finding an explanation for a family's cancer history often outweighs the potential burden of results with uncertain significance. In addition, risk estimates for some less well-established genes are derived from studies with potential ascertainment bias and small numbers of patients.^3^ Despite the limitations of large panels, they present an opportunity to identify genetic contributions to cancer in families and to offer more tailored management to patients and family members. Such information can be used to reduce cancer risk, morbidity, and mortality.

Here we report our experience from the first 10,030 consecutive patients referred for hereditary cancer panel testing at a single clinical diagnostic laboratory, providing data on the frequency of pathogenic genetic variants by gene, gene panel, and cancer history.

## Materials and Methods

We reviewed the genetic test results and clinical data from a consecutive series of 10,030 patients referred for evaluation by an NGS hereditary cancer panel between August 2013 and October 2014. Consent was obtained from all patients to have their information used in anonymized studies. Personal and family histories of cancer were provided by the referring clinician. Patients were categorized as having breast, colon, stomach, ovarian, endometrial, or pancreatic cancer; other cancer types were not singled out for analysis. We stratified patients with breast and ovarian cancer according to reported previous *BRCA1*/*2* genetic testing; for most analyses we focused on those without previous testing. Individuals with colon or stomach cancer were combined because of the small number of patients with stomach cancer.

Eight multigene cancer panels comprising combinations of 29 genes were included in this analysis (**Table 1**). Genes included in the panels had published evidence of association with hereditary cancer in at least two independent publications by two different groups. The genes were broadly grouped into three risk categories based on penetrance data available in 2012, when the tests were developed:

- High risk: *APC*, *BMPR1A*, *BRCA1*, *BRCA2*, *CDH1*, *CDKN2A*, *EPCAM*, *MLH1*, *MSH2*, *MSH6*, *MUTYH*, *PMS2*, *PTEN*, *SMAD4*, *STK11*, *TP53*, *VHL*
- Moderate risk: *ATM*, *CHEK2*, *PALB2*
- Increased but less well-defined risk: *AXIN2*, *BARD1*, *BRIP1*, *CDK4*, *FANCC*, *NBN*, *RAD51C*, *RAD51D*, *XRCC2*

Testing included NGS and exon-level array comparative genomic hybridization–based or multiplex ligation-dependent probe amplification (MLPA)–based deletion/duplication analysis of all exons and adjacent noncoding regions (see “Technical methods” below for details). Genetic variants were classified as pathogenic, likely pathogenic, VUS, likely benign, or benign/polymorphism, following the 2007 guidelines from the American College of Medical Genetic and Genomics.^4^ The overall classification of any test was based on the most severe variant reported. Reports with pathogenic or likely pathogenic variants were considered positive for many analyses in this study, and those with only benign or likely benign variants were considered negative. Because *MUTYH*-associated polyposis is an autosomal-recessive syndrome, only patients with two positive variants in *MUTYH* were counted in yield figures. All variants identified are submitted to ClinVar for public access.^5^

A subset of variants underwent reclassification since originally being reported; the variant classifications used in this analysis represent the current classifications. Providers were notified of changes in variant classifications.

### Technical methods

Genomic DNA was isolated from whole blood using a QIAsymphony DNA kit, and from oral rinse using a QIAsymphony DSP Virus/Pathogen Midi Kit (Qiagen, Valencia, CA). Genomic DNA was enriched for the complete coding region and splice-site junctions of the genes of interest using custom SureSelect targeted capture (Agilent, Santa Clara, CA). NGS and deletion/duplication analysis were performed for all coding regions as well as a portion of the 5′ untranslated region, 3′ untranslated region, and intronic regions for all the genes on each panel, with the exception of *EPCAM*, for which only deletion/duplication analysis was performed. The entire promoter region of the *PTEN* gene was also analyzed using the same methodology as for the coding regions. The products were sequenced on Illumina MiSeq or HiSeq instrument with paired-end reads (Illumina Inc., San Diego, CA). DNA sequence was mapped to a masked version of the published human genome build UCSC hg19/GRCh37 reference sequence using BWA-Mem version 0.7.8.^6,7^ Local realignment around insertion/deletion sites and regions with poor mapping quality was performed using the Genome Analysis Toolkit version 1.6 IndelRealigner.^8^ Variant calls were generated by the union of SAMtools version 0.1.18,^9^ Genome Analysis Toolkit UnifiedGenotyper,^8^ and a GeneDx-developed heuristic caller. Capillary sequencing (Applied Biosystems/Life Technologies, Grand Island, NY) on a newly extracted DNA sample was used to confirm all variants with clinical or uncertain significance and to fill in sequence for regions with fewer than 15 reads by NGS. Any potential variant position with coverage of fewer than 50 reads was reviewed by analysts and analyzed by capillary sequencing if suspect. Of cases analyzed, 75% required no capillary sequencing for additional coverage, 3.2% required capillary sequencing of one amplicon for additional coverage, 17.4% required two amplicons, and 4.4% required more than two amplicons. Long-range polymerase chain reaction was used to distinguish variants in *PMS2* from those in the *PMS2* pseudogene, *PMS2CL*. Deletion/duplication analysis was performed via custom-designed exon-targeted array comparative genomic hybridization (Agilent) or, for *STK11*, MLPA (MRC-Holland, Amsterdam, The Netherlands). Confirmation of copy-number changes detected on array comparative genomic hybridization was performed by MLPA or repeat microarray analysis, and those originally detected by MLPA were confirmed by repeat MLPA or quantitative polymerase chain reaction using the Universal Probe Library (Roche, Indianapolis, IN).

### Variant classification

We utilized the 2007 American College of Medical Genetics and Genomics framework as a guideline for variant classification.^4^ Every variant was analyzed with a four-level comprehensive review process by master's- and PhD-level analysts, board-certified genetic counselors, and PhD-level Fellow of the American College of Genetics and Genomics clinical molecular geneticists. Specific tools and resources include the Exome Sequencing Project,^10^ SIFT,^11^ PolyPhen2,^12^ MutationTaster,^13^ splice site prediction model BDGP,^14^ NetGene2,^15^ Softberry,^16^ Human Gene Mutation Database,^17^ and locus-specific databases including the Breast Cancer Information Core^18^ and InSiGHT.^19^

Evidence supporting benign classification of a variant included, but was not limited to, silent variants without evidence of splicing defects; published functional assessment with results similar to wild type; lack of cosegregation in large, published pedigrees; population frequencies that are too high to be consistent with a rare inherited cancer syndrome; and a lack of evolutionary conservation. Evidence supporting pathogenicity of a variant included, but was not limited to, truncating variants predicted to cause nonsense-mediated messenger RNA decay; published functional assessment demonstrating a clear effect on function; cosegregation with disease in large, published pedigrees; absence of the variant in population databases; evolutionary conservation across species; and location of a variant in a functional domain. Sufficient published information was required for a missense or in-frame variant to be classified as pathogenic. Variants classified as having uncertain significance were often missense for which there was a lack of published information to inform a pathogenic or benign classification, or, alternatively, conflicting data regarding clinical effect. VUSs often were not observed in population databases or were not frequent enough to be considered polymorphisms.

Lines of evidence used to inform a classification held different weights and were generally characterized into stand-alone, strong, and moderate categories. For example, since loss of function is an established mechanism of all the genes currently on the panels, variants creating a null allele fulfilled a stand-alone criterion for pathogenicity. Similarly, an allele frequency of at least 1% among sufficiently large groups within population databases was often sufficient for a benign classification. Well-established functional studies served as strong criteria for classification in many cases, and in silico models were weighted as moderate lines of evidence. Likely pathogenic variants had a high probability of being pathogenic. Variants reached the threshold for likely pathogenic but not pathogenic if, for example, functional studies revealed reduced but not absent activity or the variant was present at a low frequency among healthy controls. The literature about a particular variant is re-reviewed if the variant is observed again to ensure robust, up-to-date classifications. Information from new cases, such as the establishment of phase for two variants in the same gene, or establishing that a variant is de novo, may influence the classification of a variant.

## Results

During the first 15 months in which we offered cancer panels, 10,030 patients underwent analysis with a NGS hereditary cancer panel. Sixteen patients were tested with two different panels for a total of 10,046 panels. Patient characteristics including age at genetic testing, reported ancestry, and cancer diagnosis are summarized in **Table 2**. Over half of the individuals referred for testing were women with breast cancer (*n* = 5,209). Of those, 3,315 (63.6%) had not, to our knowledge, had previous *BRCA1*/*2* testing. Unaffected individuals comprised 25.2% of the study population.

### Frequency of pathogenic and likely pathogenic variants

Overall, 9.0% (901/10,030) of patients in this series were found to carry at least one pathogenic or likely pathogenic variant, totaling 937 variants (**Supplementary Table S1** online). Positive results were split relatively evenly between well-established genes—including *BRCA1*/*2*, Lynch syndrome, and other high-risk genes (51.8%)—and more recently described genes with moderate or unknown risk (48.2%) (**Table 3**). Likely pathogenic variants comprised 10.6% (99/937) of all positive results, with *CHEK2* accounting for the majority of all likely pathogenic variants (68.7%; 68/99). Missense variants accounted for 18.4% (172/937) of variants, whereas the remainder included splicing, gross rearrangements, frameshifts, nonsense, and regulatory variants (**Supplementary Table S2** online). Large deletions and duplications across all panels accounted for 7.0% (66/937) of positive results, composing 0.7% of the entire testing population. The majority of large rearrangements were in *BRCA1* (*n* = 15) and *MSH2* (*n* = 10).

**Figure 1:**
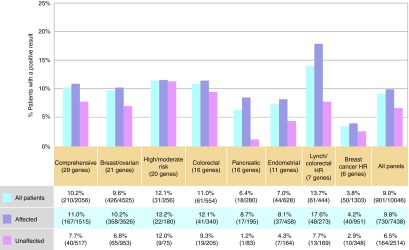
**Frequency of positive results**. A positive result includes at least one pathogenic or likely pathogenic variant. “All patients” includes the entire testing population regardless of cancer history. “Affected” includes individuals reporting any type of cancer. Clinical information was not provided for 93 patients (94 panel tests); thus the sum of those affected and those unaffected does not equal the total number tested. HR, high risk.

The yields for positive results, stratified by panel and affected status, are shown in **Figure 1**. The Lynch syndrome/colorectal cancer panel, containing *MLH1*, *MSH2*, *MSH6*, *PMS2*, *EPCAM*, *APC*, and *MUTYH*, had the highest yield (13.7% overall, 17.6% among affected individuals), whereas the breast cancer high-risk panel containing *BRCA1*, *BRCA2*, *CDH1*, *PTEN*, *STK11*, and *TP53* had the lowest yield (3.8% overall, 4.2% among affected individuals). On every panel, the positive yield was higher among affected than unaffected individuals.

### Frequency of VUSs

**Figure 2:**
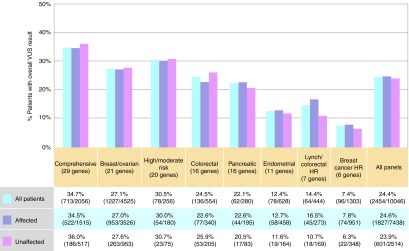
**Frequency of variant of uncertain significance (VUS) results**. This represents the proportion of patients with a VUS as the overall test result. “All patients” includes the entire testing population regardless of cancer history. “Affected” includes individuals reporting any type of cancer. Clinical information was not provided for 93 patients (94 panel tests); thus the sum of those affected and those unaffected does not equal the total number tested. HR, high risk.

While VUS as the overall test classification was not associated with affection status, it was associated with the number of genes on a panel. The highest VUS frequency was observed on the largest panel, the comprehensive panel with 29 genes, with the lowest rate on the high-risk breast cancer panel with only six genes (**Figure 2**). VUS frequencies varied by reported ancestry. Looking at the subset of individuals reporting only one ancestry, those reporting Hispanic or Caucasian ancestry had the lowest rates across all panels (20.4 and 22.7%, respectively), whereas those reporting Asian or African-American ancestry were more likely to have a VUS as the highest classification on the report (37.3 and 39.7%).

### Yield by cancer diagnosis

We also examined positive yields among individuals with the same type of cancer regardless of the panel ordered (**Table 3**). Individuals with colon/stomach cancer had the highest yield (14.8%; 113/764) of positive results. The majority of those positive results were in well-established colon cancer genes: *MLH1*, *MSH2*, *MSH6*, *PMS2*, *EPCAM*, *MUTYH*, *APC*, *PTEN*, and *STK11*. However, 28.2% (35/124) were observed in genes considered nonclassical for gastrointestinal cancers: *BRCA1*, *BRCA2*, *CHEK2*, *ATM*, *PALB2*, *BRIP1*, and *RAD51D*. *BRCA1*/*2* alone accounted for 9.7% (12/124) of positive variants identified in individuals diagnosed with colon cancer.

Focusing on breast cancer, 9.7% (320/3,315) of female breast cancer patients without prior *BRCA1*/2 testing were found to carry a pathogenic or likely pathogenic variant. *BRCA1* and *BRCA2* (with 70 and 59 variants, respectively) accounted for 39.1% of positive findings, meaning the majority of positive results identified in women with breast cancer were in genes other than *BRCA1*/*2*. Other high-risk genes, including *TP53*, *PTEN*, and *CDH1*, accounted for 5.8% (19/330) of positive results. Furthermore, 5.2% (17/330) of positive variants were in the Lynch syndrome genes.

Moderate and less well-defined risk genes accounted for 50.0% (165/330) of all positive results among women with breast cancer. The moderate-risk genes—*CHEK2*, *ATM*, and *PALB2*—had positive yields of 2.0% (*n* = 66), 1.0% (*n* = 33), and 0.8% (*n* = 25), respectively, among the 3,315 women with breast cancer and no previous testing. Two specific disease-related *CHEK2* variants, c.1100delC and p.Ile157Thr, were identified at high frequencies of 0.8 and 0.5%, respectively, in women with breast cancer.

Pathogenic variants were identified in 11.8% (6/51) of male patients with breast cancer and were found in *BRCA1*, *BRCA2*, *CHEK2*, and *PALB2*; one man was positive for both a *BRCA1* and *CHEK2* variant. Two of the six men with pathogenic variants (one *CHEK2*, one *PALB2*) reported previous negative *BRCA1*/*2* testing.

Of women with ovarian cancer without reported previous *BRCA1/2* testing, 13.4% (89/663) were positive. *BRCA1* and *BRCA2* together accounted for 50.5% of all positive findings, Lynch syndrome genes for 14.3%, and moderate or less well-defined risk genes for 33.0%.

Nearly all women who had undergone prior *BRCA1/2* testing were negative for pathogenic variants in those genes. However, seven women with breast or ovarian cancer and previous *BRCA1*/*2* testing were found to have a positive *BRCA1*/2 finding on an NGS panel in our laboratory. Four of these women knew of their positive status before testing at our laboratory but chose to pursue additional panel testing because of bilineal familial risk or the presence of potentially heritable cancers other than breast and ovarian in the family. The other three had undergone *BRCA1*/*2* testing that would not have detected the variant they carried; previous testing included Ashkenazi Jewish founder testing, sequencing only, and sequencing plus testing for common *BRCA1* rearrangements.

Of the 453 women with endometrial cancer, the positive yield was 11.9% (*n* = 54); 7.3% (*n* = 33) of these were within a Lynch gene, most commonly *MSH6*. *CHEK2* also had a significant number of positives (*n* = 7), with an overall frequency of 1.5%. Likewise, six positive results were identified in *BRCA1*/*2*, or 10.9% (6/55) of all positive variants identified. Among 190 pancreatic cancer patients, the positive yield was 10.5% (*n* = 20), and positive results were most commonly identified in *ATM* (40.0%; 8/20), *BRCA2* (25.0%; 5/20), and *PALB2* (15.0%; 3/20).

### Multiple positive findings

Of 901 positive patients, 28 (3.1%) had more than one positive finding, reflecting 0.3% (28/10,030) of the total testing population (**Supplementary Table S3** online). Five had positive results in two highly penetrant genes, 12 had one positive result in a high-risk gene and one in a gene with moderate or unknown risk, and 11 had two positive findings in genes with moderate or unknown risk. Twenty individuals had two pathogenic variants, seven had one pathogenic and one likely pathogenic, and one had two likely pathogenic variants (*MSH6* and *CHEK2*). Twelve women with breast cancer had multiple positive findings: one with findings in two high-risk genes (*BRCA2*/*VHL*), seven with a result in one high-risk gene (e.g., *BRCA1*/*PALB2*, *BRCA2*/*CHEK2*, biallelic *MUTYH*/*CHEK2*), and four with positive results in genes of moderate or unknown risk (all *CHEK2*/*ATM* or compound heterozygous or homozygous for *CHEK2*). Of the 28 individuals with multiple positive findings, 6 (21.4%) reported multiple primary cancers.

### Findings in *TP53* and *CDH1*

Six of the 18 patients (33%) with positive findings in *TP53* did not meet classic Li-Fraumeni syndrome,^20^ Li-Fraumeni-like,^21^ 2009 Chompret,^22,23^ or National Comprehensive Cancer Network guideline criteria for *TP53* testing,^24^ resulting in a frequency of 0.06% (6/9,605) unanticipated positive results. Four patients in our cohort had a positive *CDH1* result, two of whom did not meet International Gastric Cancer Linkage Consortium testing criteria,^25^ resulting in a frequency of 0.02% (2/8,708) positive *CDH1* results in individuals who do not meet clinical criteria among those tested for the gene.

## Discussion

Before the advent of NGS, patients who underwent germ-line genetic testing for hereditary cancer were typically tested for a limited number of genes that were strongly associated with a single hereditary cancer syndrome, such as *BRCA1*/*2* or the Lynch genes. In the current era of NGS there is an opportunity to provide more information on a large number of genes, allowing for more accurate risk stratification and tailored cancer care. We provide data for over 10,000 patients who have undergone germ-line cancer genetic testing to begin to evaluate the overall yield of gene panels by cancer type and to identify the specific genes with the highest yields.

In this study population, the Lynch syndrome/colorectal cancer panel had the highest yield among all panels despite the relatively small number of genes (13.7% among all patients, 18.7% among affected patients). This high yield is likely a result of the well-established association of all genes on this panel with colorectal cancer, as well as the specific clinical history or tumor characteristics (microsatellite instability and/or immunohistochemistry) that prompted providers to order this more focused panel.

Among patients with specific cancers, yields were 9.7, 13.4, and 14.8% in patients with breast, ovarian, and colon/stomach cancer, respectively (the patients with breast or ovarian cancer did not report previous *BRCA1*/*2* testing). Importantly, a significant proportion of the positive results were in genes that are not typically associated with the referring diagnosis. For instance, 5.8% of positive results among women with breast cancer were in highly penetrant genes other than *BRCA1*/*2*. The yield in Lynch genes among breast cancer patients was 0.5% (17/3,315), higher than a published upper estimate of the prevalence of Lynch syndrome among the general population (0.2%).^26^ In addition, more than a quarter of patients with colon cancer tested positive for genes that are not considered to be classic colorectal cancer genes; *BRCA1*/*2* alone accounted for 9.7% of pathogenic variants identified in individuals with colon cancer. Similarly, over 11% of the positive findings among women with endometrial cancer were in *BRCA1*/*2*. Finally, a small number of patients whose personal and family histories were not suggestive of Li-Fraumeni syndrome were nonetheless positive for pathogenic variants in the highly penetrant *TP53* gene. Together, these findings illustrate the utility of large panels in identifying pathogenic variants in high-risk genes that might not have been considered based only on a patient's personal cancer history. In some of the cases described above, the family history might have prompted broader testing and uncovered the variant identified on the larger panel, but this is not true for all examples.

### Genes with moderate or less well-defined risk

Notably, almost half (48.2%) of positive variants identified in our overall testing population were in genes with moderate or undefined risk. While literature supports an association with cancer, the magnitude of the risk and complete cancer spectrum for variants in these genes is unclear. As Easton et al.^3^ convey, establishing an accurate risk requires studies that are sufficiently large and without ascertainment bias, and not many of these have been performed. Positive results in genes with moderate or unknown risk can present quandaries for providers given the lack of established guidelines for medical management. Furthermore, it often is not clear whether the identified variant in these genes is the sole genetic cause of the cancer in the family.

Pathogenic variants in *CHEK2*, *ATM*, and *PALB2* are thought to confer a breast cancer risk that is above the 20% lifetime threshold for which breast magnetic resonance imaging is considered.^24,27^ Thus identifying pathogenic variants in any of these three genes in particular can have clear implications for clinical management.

*CHEK2* is well established as a moderate-risk susceptibility gene for breast cancer, with weaker associations reported for colon and other cancers.^28,29,30^ The high yield in *CHEK2* among individuals with all types of cancer may be in part a result of the high population frequency of the common *CHEK2* disease-related variants. *CHEK2* c.1100delC is present in up to 1% in Northern Europeans, whereas p.Ile157Thr is found in 1.4% in the 1000 Genomes Project and up to 5% of individuals of Finnish or Polish ancestry.^31^ Even in our population with multiple ancestries, *CHEK2* c.1100delC and p.Ile157Thr comprised notable proportions of positive findings, with overall frequencies of 0.8 and 0.5%, respectively. The positive yield in *CHEK2* among women with breast cancer (2.0%; 66/3,315) or endometrial cancer (1.5%; 7/453) were consistent with published frequencies.^29,32,33^

*PALB2* is emerging as a gene that plays a major role in inherited breast cancer; recent data suggest a lifetime risk for *PALB2* carriers ranging from 33 to 58%, depending on the family history of cancer.^34^ If these risks are confirmed in subsequent studies, *PALB2* would impart the same level of breast cancer risk as *BRCA2* and therefore may warrant similar breast cancer medical management recommendations. *PALB2* alone accounted for 7.6% (25/330) of all positive findings among female patients with breast cancer without previous testing, which draws attention to its relevance in breast cancer genetic testing. Among the male patients with breast cancer, five of six who tested positive had a variant in *CHEK2* or *PALB2*, both of which have been previously implicated in male breast cancer.^35,36^ *ATM* accounted for 10.0% (33/330) of positive findings among female patients with breast cancer, making it a common finding.

### Variants of uncertain significance

Although VUSs are not considered clinically actionable, they can create concern for patients and clinicians. The observed VUS frequency was correlated with the number of genes on each panel, related in part to the fact that many of the larger panels have more newly characterized genes. Less well-studied genes, by definition, contribute to higher VUS rates since few missense variants in those genes have been functionally characterized or studied in families. VUS frequencies also varied by reported ancestry and were highest among individuals of African American and Asian ancestry. This is related, in part, to the higher proportion of Caucasians included in reference databases.

During the course of performing NGS cancer panels, the VUS frequencies at our laboratory varied by panel, decreasing modestly by a few percentage points on the breast/ovarian cancer, Lynch syndrome/colorectal cancer, and high/moderate risk panels. Approximately 200 variants (2.6% of all reported variants) have been reclassified since they were originally reported. The majority (~90%) of reports that were revised were done so because of a variant being downgraded from uncertain to likely benign, often because of increasing numbers of observations in the testing population or in recently available large reference data sets.

### Multiple positive findings

Previously, once a pathogenic variant was identified in a hereditary cancer gene, additional testing was rarely performed because it was often assumed that only that one pathogenic variant was necessary to account for the cancer. However, additional pathogenic variants in genes that were never previously tested may also contribute to cancer risk in the family. Current estimates suggest that up to 3% of individuals who test positive on a multigene cancer test have more than one pathogenic finding, and our frequency of 3.1% is consistent with these estimates.^1,2,30^ In our population, a second positive variant would have been missed in 23 of 28 patients if individuals had been tested only for established high-penetrance genes such as *BRCA1*/*2* or the Lynch syndrome genes. Notably, over 20% of individuals with two positive variants reported multiple primary malignancies. Whether individuals with multiple primary tumors are more likely to carry more than one positive variant is a hypothesis that needs to be examined in future studies.

### Unexpected findings in highly penetrant genes

There is concern among providers about identifying a disease-causing variant in a high-penetrance gene in a family that does not have the classically associated phenotype. Two genes of greatest concern are *TP53* (Li-Fraumeni syndrome) and *CDH1* (hereditary diffuse gastric cancer), both of which are associated with high risks of cancer and management options that include prophylactic surgery and/or extensive, though often inadequate, surveillance.^24,25^ If a family without a classic phenotype is found to have a positive finding in either of these genes, the providers ordering the testing may question the appropriateness of applying standard management guidelines.

Two individuals in our referral population who were found to carry *CDH1* variants (one pathogenic, one likely pathogenic) did not meet *CDH1* testing guidelines. However, their phenotypes were not outside of the *CDH1* spectrum; one had diffuse gastric cancer and the other had breast cancer with lobular features plus a family history of lobular breast cancer. By contrast, of the six individuals found to carry pathogenic or likely pathogenic *TP53* variants who did not meet testing criteria for *TP53*, three did not have phenotypes characteristic of Li-Fraumeni syndrome. Those with suspicious histories (bilateral breast cancer at age 42; breast cancer at age 54 and sarcoma at age 56; a strong family history of early-onset colon cancer) all carried pathogenic variants. The three remaining individuals—two with pathogenic variants and one with a likely pathogenic *TP53* variant—had cancer at older ages and family histories that are not typical of Li-Fraumeni syndrome. While de novo events may explain weaker family histories in some cases, these findings may also demonstrate our evolving understanding of syndrome phenotypes and penetrance associated with *CDH1* and *TP53*. In sum, panel testing identified variants in high-risk genes that might not otherwise have been targeted, although the overall risk for an unexpected finding in either of these two genes was low for any given patient undergoing testing.

### Limitations

As a testing laboratory, the clinical information we report is limited to what is provided by the referring clinician. Age at diagnosis, pathology, and complete family history were not available for every case and were not used in this analysis. Furthermore, to our knowledge, individuals included in the analyses represent unrelated probands, but two or more family members might have been referred for a panel test. Finally, the small number of patients with certain cancers, and the fact that not all individuals referred for testing were tested for the same group of genes, suggest the need for replication. However, the large number of patients tested overall provides important data on the frequency of pathogenic/likely pathogenic variants as well as variants of uncertain significance that are likely to be encountered in routine clinical practice.

### Conclusion

We report the largest series to date of patients undergoing NGS for hereditary cancer gene panels. Our experience demonstrates that multigene panels have the potential to identify pathogenic variants in genes that would not typically have been tested, both in high-risk genes not associated with a patient's personal cancer history and in genes with moderate or less well-defined penetrance which, before the availability of multigene panels, were not often tested. The high frequency of positive findings in these more recently identified cancer genes underscores their potential contribution to hereditary cancer risk and evolving impact on medical management. Not all patients or providers are comfortable dealing with uncertainty in management, and expansive gene panels including genes with currently unknown risks are not appropriate in all clinical contexts or for all patients. Many patients and providers, however, would like to have the information about these genes so that as more information becomes available they are prepared to act immediately. Our study provides important empirical data to inform clinical decision making when choosing between single genes and NGS cancer panel testing in a variety of clinical scenarios.

## Disclosure

The following individuals are employed by GeneDx/BioReference Labs and have salary as the only disclosure: L.R.S., M.L.M., R.N., K.J.V.P., S.M.W., L.Y., E.M.V., J.B., J.K.B., M.L.C., F.G., P.D.M., D.E.P.-A., G.D.P., Z.X., R.T.K., K.S.H. In addition, G.R. and S.B. hold stock of and are employed by GeneDx/BioReference Labs, and W.K.C. holds stock and has a consulting agreement with GeneDx/BioReference Labs.

## Figures and Tables

**Table 1:**
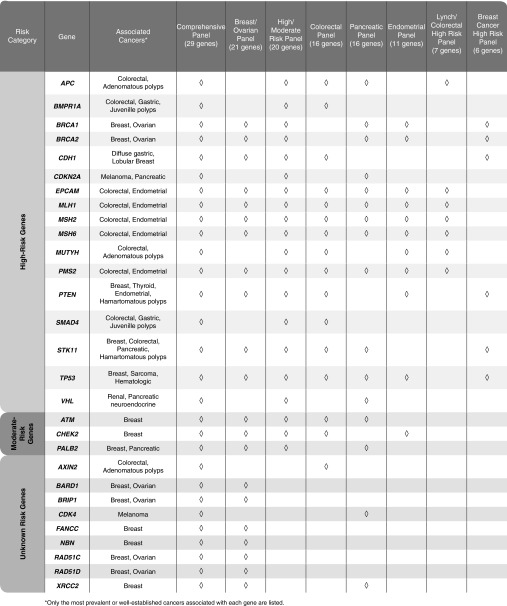
Next-generation sequencing cancer panels

**Table 2:**
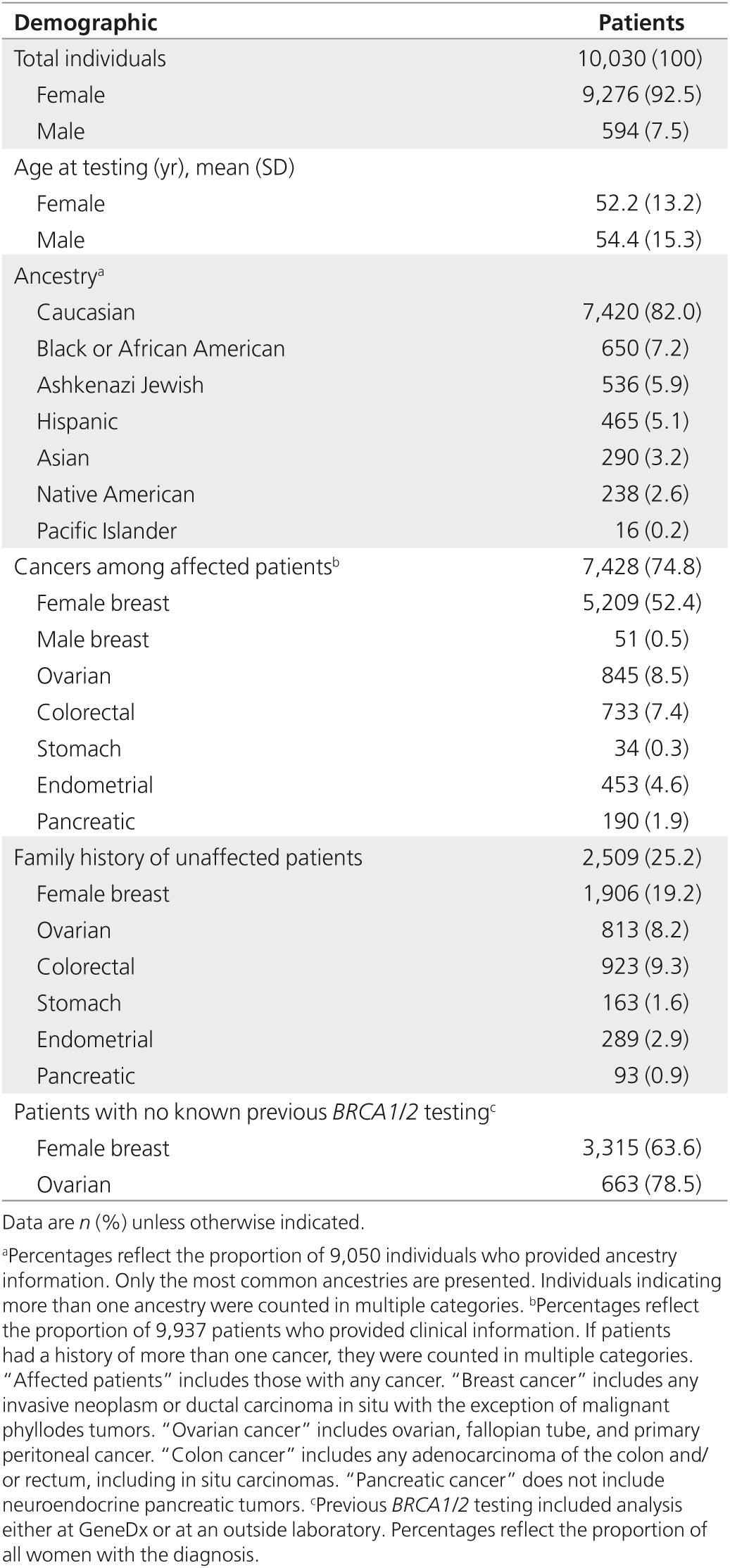
Demographics of individuals tested with a next-generation sequencing hereditary cancer panel

**Table 3:**
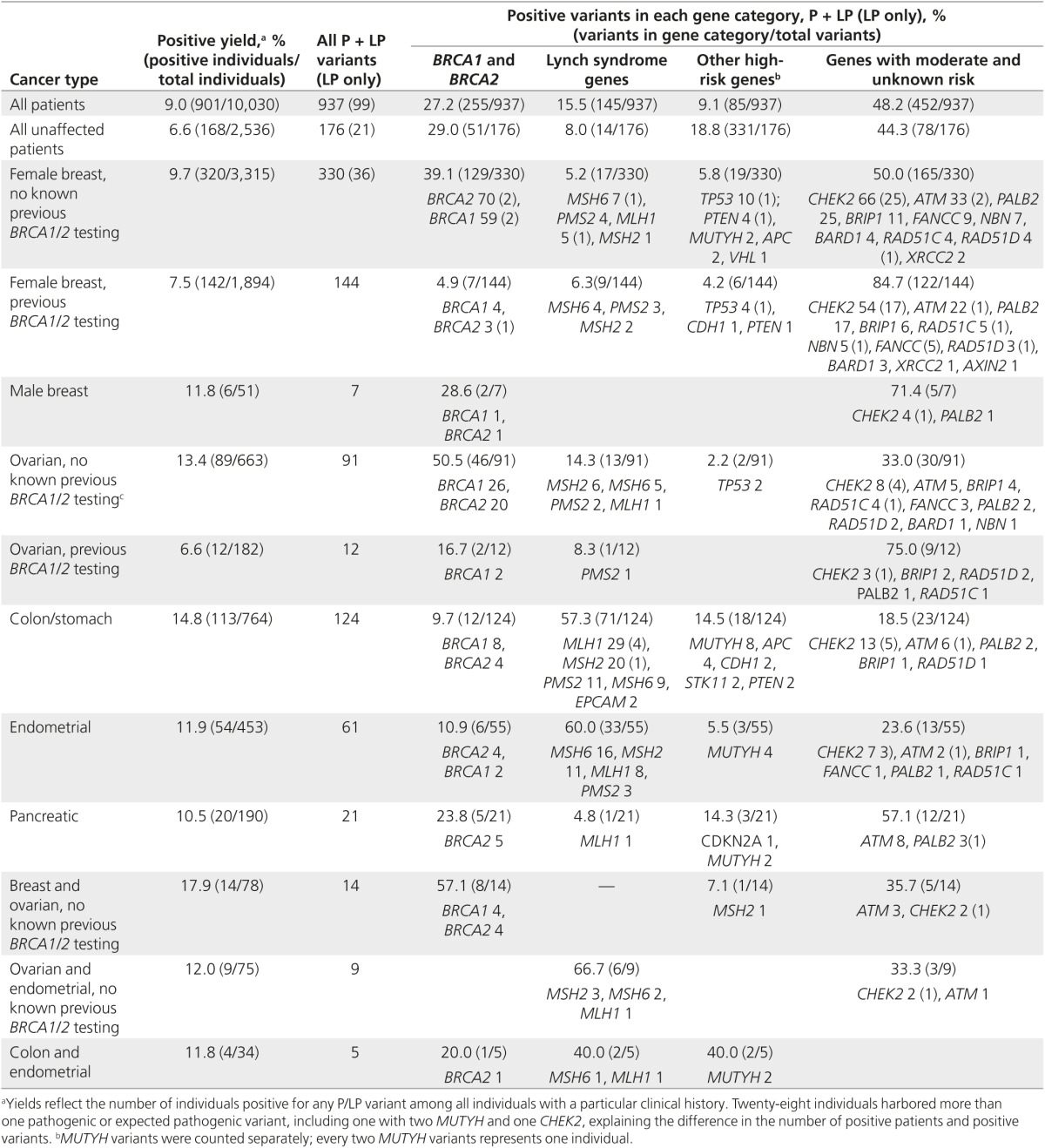
Yields by cancer type and genes with pathogenic (P) and likely pathogenic (LP) variants among patients with specific clinical histories
