# Supplementary material for: Pathogenic and likely pathogenic variant prevalence among the first 10,000 patients referred for next-generation cancer panel testing
Source: Genet Med. 2015 Dec 17;18(8):823–32. doi: 10.1038/gim.2015.166 (PMC4985612; doi:10.1038/gim.2015.166)
Supplement: Supplementary Table S1 [file gim2015166x1.doc]

| **Gene** | **Coding DNA*^a^*** | **Protein** | **Classification** | **Molecular Effect*^b^*** | **Clinical History*^c^*** | **Observations** | **Met *CDH1* or *TP53* Criteria?^d^** |
| --- | --- | --- | --- | --- | --- | --- | --- |
| *APC* | c.1192_1193delAA | p.Lys398GlufsX5 | Pathogenic | Frameshift | Polyposis | 1 |  |
| *APC* | c.1213C>T | p.Arg405Ter | Pathogenic | Nonsense | Colon polyps | 1 |  |
| *APC* | c.1312+3_1312+4delAT | na | Pathogenic | Splicing | Polyposis | 1 |  |
| *APC* | c.147_150delACAA | p.Lys49AsnfsX20 | Pathogenic | Frameshift | Colon polyps | 1 |  |
| *APC* | c.1987C>T | p.Gln663Ter | Pathogenic | Nonsense | Colon, Thyroid, Colon polyps | 1 |  |
| *APC* | c.2004delC | p.Leu669Ter | Pathogenic | Nonsense | Polyposis | 1 |  |
| *APC* | c.288T>A | p.Tyr96Ter | Pathogenic | Nonsense | Colon polyps | 1 |  |
| *APC* | c.3088A>T | p.Lys1030Ter | Pathogenic | Nonsense | Polyposis | 1 |  |
| *APC* | c.3472A>T | p.Arg1158Ter | Pathogenic | Nonsense | Gastric, Colon polyps | 1 |  |
| *APC* | c.3473_3474dupGA | p.Pro1159AspfsX7 | Pathogenic | Frameshift | Breast, Polyposis | 1 |  |
| *APC* | c.3602C>G | p.Ser1201Ter | Pathogenic | Nonsense | Colon polyps | 1 |  |
| *APC* | c.426_427delAT | p.Leu143AlafsX4 | Pathogenic | Frameshift | Polyposis | 1 |  |
| *APC* | c.4875delA | p.Gln1625HisfsX25 | Pathogenic | Frameshift | Breast, Colon polyps | 1 |  |
| *APC* | c.531+5G>A | na | Pathogenic | Splicing | Colon polyps | 1 |  |
| *APC* | c.5490_5493delTGAA | p.Asn1830LysfsX32 | Pathogenic | Frameshift | Colon polyps | 1 |  |
| *APC* | c.5803delC | p.Gln1935SerfsX35 | Pathogenic | Frameshift | Colon, Gastric, Colon polyps | 1 |  |
| *APC* | c.5844_5850  delTGAAAAGinsGGAAAA | p.Asp1948GlufsX22 | Pathogenic | Frameshift | Polyposis | 1 |  |
| *APC* | c.5936_5939delACAA | p.Asn1979ThrfsX64 | Pathogenic | Frameshift | Colon polyps | 1 |  |
| *APC* | c.637C>T | p.Arg213Ter | Pathogenic | Nonsense | Colon, Colon polyps | 1 |  |
| *APC* | c.6383delC | p.Ala2128ValfsX11 | Pathogenic | Frameshift | Colon | 1 |  |
| *APC* | Deletion of Entire *APC* Gene | na | Pathogenic | Gross deletion | Hematologic | 1 |  |
| *ATM* | c.1235G>A | p.Trp412Ter | Pathogenic | Nonsense | None | 1 |  |
| *ATM* | c.1339C>T | p.Arg447Ter | Pathogenic | Nonsense | Ovarian | 1 |  |
| *ATM* | c.154G>T | p.Gly52Ter | Pathogenic | Nonsense | None | 1 |  |
| *ATM* | c.1564_1565delGA | p.Glu522IlefsX43 | Pathogenic | Frameshift | Breast, Melanoma, Pancreatic | 7 |  |
| *ATM* | c.170G>A | p.Trp57Ter | Pathogenic | Nonsense | Colon | 1 |  |
| *ATM* | c.2250G>A | na | Expected Pathogenic | Splicing | Breast, Endometrial, Colon polyps | 1 |  |
| *ATM* | c.2251-10T>G | na | Pathogenic | Splicing | Breast | 1 |  |
| *ATM* | c.2376+1G>T | na | Pathogenic | Splicing | None | 1 |  |
| *ATM* | c.237delA | p.Lys79AsnfsX37 | Pathogenic | Frameshift | Ovarian | 1 |  |
| *ATM* | c.2502dupA | p.Val835SerfsX7 | Pathogenic | Frameshift | Breast, Melanoma | 2 |  |
| *ATM* | c.2564dupT | p.Met855IlefsX5 | Pathogenic | Frameshift | None | 1 |  |
| *ATM* | c.2638+2T>C | na | Pathogenic | Splicing | Breast | 1 |  |
| *ATM* | c.2880delC | p.Leu961CysfsX10 | Pathogenic | Frameshift | Breast | 1 |  |
| *ATM* | c.2897_2899  delTTCinsGCCAA | p.Val966GlyfsX6 | Pathogenic | Frameshift | Breast | 1 |  |
| *ATM* | c.3049C>T | p.Gln1017Ter | Pathogenic | Nonsense | Breast | 1 |  |
| *ATM* | c.3154-2A>G | na | Pathogenic | Splicing | None | 1 |  |
| *ATM* | c.3245_3247  delATCinsTGAT | p.His1082LeufsX14 | Pathogenic | Frameshift | Breast, Pilocytic astrocytoma | 2 |  |
| *ATM* | c.3304G>T | p.Gly1102Ter | Pathogenic | Nonsense | Breast | 1 |  |
| *ATM* | c.3372C>G | p.Tyr1124Ter | Pathogenic | Nonsense | Breast, Pulmonary carcinoid | 1 |  |
| *ATM* | c.3526delC | p.Leu1176CysfsX5 | Pathogenic | Frameshift | Hematologic | 1 |  |
| *ATM* | c.368delA | p.Tyr123LeufsX6 | Pathogenic | Frameshift | Breast | 1 |  |
| *ATM* | c.3747-1G>C | na | Pathogenic | Splicing | None | 1 |  |
| *ATM* | c.3802delG | p.Val1268Ter | Pathogenic | Nonsense | Breast | 1 |  |
| *ATM* | c.3931C>T | p.Gln1311Ter | Pathogenic | Nonsense | Breast, Ovarian | 1 |  |
| *ATM* | c.4143dupT | p.Pro1382SerfsX6 | Pathogenic | Frameshift | Pancreatic | 1 |  |
| *ATM* | c.4373delG | p.Gly1458GlufsX15 | Pathogenic | Frameshift | None | 1 |  |
| *ATM* | c.4394T>C | p.Leu1465Pro | Expected Pathogenic | Missense | Esophageal, Gastric | 1 |  |
| *ATM* | c.4625dupT | p.Leu1542PhefsX8 | Pathogenic | Frameshift | Breast | 1 |  |
| *ATM* | c.5201_5202insAT | p.Thr1735LeufsX4 | Pathogenic | Frameshift | Colon polyps | 1 |  |
| *ATM* | c.5290delC | p.Leu1764TyrfsX12 | Pathogenic | Frameshift | Breast | 1 |  |
| *ATM* | c.5320-5_5320-2delTCTA | na | Pathogenic | Splicing | Breast | 1 |  |
| *ATM* | c.538C>T | p.Gln180Ter | Pathogenic | Nonsense | Breast | 1 |  |
| *ATM* | c.549_550delTA | p.His183GlnfsX6 | Pathogenic | Frameshift | Colon | 1 |  |
| *ATM* | c.5712dupA | p.Ser1905IlefsX25 | Pathogenic | Frameshift | Pancreatic | 1 |  |
| *ATM* | c.5784dupT | p.Asn1929Ter | Pathogenic | Nonsense | Breast | 1 |  |
| *ATM* | c.5791delGinsCCT | p.Ala1931ProfsX7 | Pathogenic | Frameshift | Melanoma, Pancreatic | 1 |  |
| *ATM* | c.5932G>T | p.Glu1978Ter | Pathogenic | Nonsense | Breast, Melanoma, Ovarian | 2 |  |
| *ATM* | c.6095G>A | na | Pathogenic | Splicing | Pancreatic | 1 |  |
| *ATM* | c.6100C>T | p.Arg2034Ter | Pathogenic | Nonsense | Breast, Endometrial, Peritoneal, Thyroid | 1 |  |
| *ATM* | c.640delT | p.Ser214ProfsX16 | Pathogenic | Frameshift | None | 1 |  |
| *ATM* | c.6572+1G>A | na | Pathogenic | Splicing | None | 1 |  |
| *ATM* | c.6679C>T | p.Arg2227Cys | Expected Pathogenic | Missense | Breast | 1 |  |
| *ATM* | c.6976-10_6989del24 | na | Pathogenic | Splicing | Breast, Pancreatic | 2 |  |
| *ATM* | c.6976-2A>C | na | Pathogenic | Splicing | Colon, Polyposis | 1 |  |
| *ATM* | c.7181C>T | p.Ser2394Leu | Expected Pathogenic | Missense | None | 1 |  |
| *ATM* | c.7271T>G | p.Val2424Gly | Pathogenic | Missense | Breast, Melanoma, Ocular melanoma, Thyroid | 7 |  |
| *ATM* | c.742C>T | p.Arg248Ter | Pathogenic | Nonsense | Breast | 1 |  |
| *ATM* | c.7456C>T | p.Arg2486Ter | Pathogenic | Nonsense | Breast | 1 |  |
| *ATM* | c.7463G>A | p.Cys2488Tyr | Expected Pathogenic | Missense | None | 1 |  |
| *ATM* | c.7630-2A>C | na | Pathogenic | Splicing | Breast | 2 |  |
| *ATM* | c.7638_7646  delTAGAATTTC | p.Arg2547_Ser2549del | Pathogenic | In-frame deletion | Breast | 2 |  |
| *ATM* | c.7788G>A | na | Pathogenic | Splicing | None | 1 |  |
| *ATM* | c.7838_7839dupGA | p.Pro2614AspfsX18 | Pathogenic | Frameshift | Breast | 1 |  |
| *ATM* | c.7913G>A | p.Trp2638Ter | Pathogenic | Nonsense | Colon | 1 |  |
| *ATM* | c.7998dupT | p.Met2667TyrfsX4 | Pathogenic | Frameshift | Breast, Gastric | 1 |  |
| *ATM* | c.8147T>C | p.Val2716Ala | Expected Pathogenic | Missense | Breast | 1 |  |
| *ATM* | c.8264_8268delATAAG | p.Tyr2755CysfsX12 | Pathogenic | Frameshift | Breast, Colon polyps | 1 |  |
| *ATM* | c.8266A>T | p.Lys2756Ter | Pathogenic | Nonsense | Breast | 2 |  |
| *ATM* | c.8418+5_8418+8delGTGA | na | Pathogenic | Splicing | Breast | 1 |  |
| *ATM* | c.8494C>T | p.Arg2832Cys | Pathogenic | Missense | Breast | 1 |  |
| *ATM* | c.8565_8566delTGinsAA | p.Ser2855_Val2856  delinsArgIle | Pathogenic | In-frame indel | Breast | 2 |  |
| *ATM* | c.8786+1G>A | na | Pathogenic | Splicing | Breast | 3 |  |
| *ATM* | c.9022C>T | p.Arg3008Cys | Pathogenic | Missense | Breast | 1 |  |
| *ATM* | c.9112delC | p.Gln3038ArgfsX3 | Pathogenic | Frameshift | Breast | 1 |  |
| *ATM* | Deletion exons 27-59 | na | Pathogenic | Gross deletion | Pancreatic | 1 |  |
| *ATM* | Deletion exons 57-63 | na | Pathogenic | Gross deletion | Astrocytoma, Glioblastoma, Colon polyps | 2 |  |
| *ATM* | Deletion exons 62-63 | na | Pathogenic | Gross deletion | Breast | 1 |  |
| *AXIN2* | c.-12_8del20 | na | Pathogenic | Loss of initiation codon | Breast | 1 |  |
| *BARD1* | c.1652C>G | p.Ser551Ter | Pathogenic | Nonsense | None | 1 |  |
| *BARD1* | c.1690C>T | p.Gln564Ter | Pathogenic | Nonsense | Breast, Ovarian | 2 |  |
| *BARD1* | c.1935_1954dup20 | p.Glu652ValfsX69 | Pathogenic | Frameshift | Breast | 2 |  |
| *BARD1* | c.1996C>T | p.Gln666Ter | Pathogenic | Nonsense | Breast | 1 |  |
| *BARD1* | c.3G>A | p.Met1? | Pathogenic | Loss of initiation codon | Breast | 1 |  |
| *BARD1* | c.448C>T | p.Arg150Ter | Pathogenic | Nonsense | Breast | 1 |  |
| *BARD1* | c.607G>T | p.Gly203Ter | Pathogenic | Nonsense | None | 1 |  |
| *BARD1* | c.623dupA | p.Lys209GlufsX5 | Pathogenic | Frameshift | Breast | 1 |  |
| *BMPR1A* | c.262G>T | p.Glu88Ter | Pathogenic | Nonsense | Colon polyps | 1 |  |
| *BMPR1A* | c.369delA | p.Glu123AspfsX21 | Pathogenic | Frameshift | Polyposis | 1 |  |
| *BMPR1A* | Deletion exon 3 | na | Pathogenic | Gross deletion | Colon polyps | 1 |  |
| *BRCA1* | c.1116G>A | p.Trp372Ter | Pathogenic | Nonsense | Melanoma | 1 |  |
| *BRCA1* | c.1175_1214del40 | p.Leu392GlnfsX5 | Pathogenic | Frameshift | Bladder, Breast, Colon, Hematologic, Male Breast, Prostate, Urethral, Colon polyps | 3 |  |
| *BRCA1* | c.1360_1361delAG | p.Ser454Ter | Pathogenic | Nonsense | Ovarian | 1 |  |
| *BRCA1* | c.1687C>T | p.Gln563Ter | Pathogenic | Nonsense | Ovarian | 1 |  |
| *BRCA1* | c.1812delA | p.Ala605HisfsX7 | Pathogenic | Frameshift | Breast, Colon | 1 |  |
| *BRCA1* | c.181T>G | p.Cys61Gly | Pathogenic | Missense | Breast, Thyroid | 4 |  |
| *BRCA1* | c.1860delT | p.His621MetfsX5 | Pathogenic | Frameshift | Ovarian | 1 |  |
| *BRCA1* | c.1953_1956delGAAA | p.Lys653SerfsX47 | Pathogenic | Frameshift | Breast | 1 |  |
| *BRCA1* | c.1961dupA | p.Tyr655ValfsX18 | Pathogenic | Frameshift | Breast | 1 |  |
| *BRCA1* | c.2035A>T | p.Lys679Ter | Pathogenic | Nonsense | Ovarian | 3 |  |
| *BRCA1* | c.213-11T>G | na | Pathogenic | Splicing | Breast | 1 |  |
| *BRCA1* | c.213-12A>G | na | Pathogenic | Splicing | None | 1 |  |
| *BRCA1* | c.2389G>T | p.Glu797Ter | Pathogenic | Nonsense | Breast | 1 |  |
| *BRCA1* | c.2411_2412delAG | p.Gln804LeufsX5 | Pathogenic | Frameshift | Breast | 1 |  |
| *BRCA1* | c.2457delC | p.Asp821IlefsX25 | Pathogenic | Frameshift | Breast | 2 |  |
| *BRCA1* | c.2603C>G | p.Ser868Ter | Pathogenic | Nonsense | Ovarian | 1 |  |
| *BRCA1* | c.2681_2682delAA | p.Lys894ThrfsX8 | Pathogenic | Frameshift | Colorectal | 1 |  |
| *BRCA1* | c.2722G>T | p.Glu908Ter | Pathogenic | Nonsense | Breast | 2 |  |
| *BRCA1* | c.2767_2770delGTTA | p.Val923IlefsX76 | Pathogenic | Frameshift | Endometrial | 1 |  |
| *BRCA1* | c.2866_2870delTCTCA | p.Ser956ValfsX13 | Pathogenic | Frameshift | Breast | 1 |  |
| *BRCA1* | c.2934T>G | p.Tyr978Ter | Pathogenic | Nonsense | Peritoneal | 1 |  |
| *BRCA1* | c.2995_2996delCTinsTA | p.Leu999Ter | Pathogenic | Nonsense | None | 1 |  |
| *BRCA1* | c.303T>G | p.Tyr101Ter | Pathogenic | Nonsense | Breast | 1 |  |
| *BRCA1* | c.3178G>T | p.Glu1060Ter | Pathogenic | Nonsense | Ovarian | 1 |  |
| *BRCA1* | c.3481_3491del11 | p.Glu1161PhefsX3 | Pathogenic | Frameshift | Breast | 2 |  |
| *BRCA1* | c.3485delA | p.Asp1162ValfsX48 | Pathogenic | Frameshift | Ovarian | 1 |  |
| *BRCA1* | c.3531delT | p.Phe1177LeufsX33 | Pathogenic | Frameshift | None | 1 |  |
| *BRCA1* | c.3598C>T | p.Gln1200Ter | Pathogenic | Nonsense | Breast | 2 |  |
| *BRCA1* | c.3668_3671dupTTCC | p.Cys1225SerfsX10 | Pathogenic | Frameshift | Breast | 1 |  |
| *BRCA1* | c.3748G>T | p.Glu1250Ter | Pathogenic | Nonsense | Breast, Colon | 4 |  |
| *BRCA1* | c.3756_3759delGTCT | p.Ser1253ArgfsX10 | Pathogenic | Frameshift | Breast, Gastric, Ovarian, Skin, Colon polyps | 3 |  |
| *BRCA1* | c.3764dupA | p.Asn1255LysfsX12 | Pathogenic | Frameshift | Breast | 1 |  |
| *BRCA1* | c.3908dupT | p.Leu1303PhefsX27 | Pathogenic | Frameshift | None | 1 |  |
| *BRCA1* | c.4035delA | Glu1346LysfsX20 | Pathogenic | Frameshift | None | 1 |  |
| *BRCA1* | c.4096+1G>A | na | Pathogenic | Splicing | None | 1 |  |
| *BRCA1* | c.4183C>T | p.Gln1395Ter | Pathogenic | Nonsense | Ovarian | 2 |  |
| *BRCA1* | c.427G>T | p.Glu143Ter | Pathogenic | Nonsense | Breast, Esophageal, Colon polyp | 2 |  |
| *BRCA1* | c.4327C>T | p.Arg1443Ter | Pathogenic | Nonsense | Breast, Ovarian, Skin | 2 |  |
| *BRCA1* | c.4357+1G>A | na | Pathogenic | Splicing | Breast | 1 |  |
| *BRCA1* | c.4689C>G | p.Tyr1563Ter | Pathogenic | Nonsense | Breast | 2 |  |
| *BRCA1* | c.4868C>G | na | Expected Pathogenic | Splicing | Ovarian | 1 |  |
| *BRCA1* | c.4964_4982del19 | p.Ser1655TyrfsX16 | Pathogenic | Frameshift | Breast, Ovarian, Medullary thyroid | 1 |  |
| *BRCA1* | c.4987-1G>A | na | Pathogenic | Splicing | Breast | 1 |  |
| *BRCA1* | c.4987-2A>G | na | Pathogenic | Splicing | Breast, Ovarian | 1 |  |
| *BRCA1* | c.5095C>T | p.Arg1699Trp | Pathogenic | Missense | Peritoneal | 2 |  |
| *BRCA1* | c.5096G>A | p.Arg1699Gln | Expected Pathogenic | Missense | Breast | 1 |  |
| *BRCA1* | c.5123C>A | p.Ala1708Glu | Pathogenic | Missense | Breast | 1 |  |
| *BRCA1* | c.514delC | p.Gln172AsnfsX62 | Pathogenic | Frameshift | Breast | 1 |  |
| *BRCA1* | c.5177_5180delGAAA | p.Arg1726LysfsX3 | Pathogenic | Frameshift | Breast | 2 |  |
| *BRCA1* | c.5193+1G>T | na | Pathogenic | Splicing | Peritoneal | 1 |  |
| *BRCA1* | c.5246C>G | p.Pro1749Arg | Pathogenic | Missense | Gastric, Ovarian | 1 |  |
| *BRCA1* | c.5251C>T | p.Arg1751Ter | Pathogenic | Nonsense | Breast | 1 |  |
| *BRCA1* | c.5266dupC | p.Gln1756ProfsX74 | Pathogenic | Frameshift | Breast, Endometrial, Fallopian tube, Ovarian, Skin, Colon polyps | 15 |  |
| *BRCA1* | c.5277+1G>A | na | Pathogenic | Splicing | Breast | 2 |  |
| *BRCA1* | c.5278-1G>T | na | Pathogenic | Splicing | Breast, Lipoma | 1 |  |
| *BRCA1* | c.5324T>G | p.Met1775Arg | Pathogenic | Missense | None | 1 |  |
| *BRCA1* | c.5359_5363delTGTGGinsAGTGA | p.Cys1787_Gly1788delinsSerAsp | Pathogenic | In-frame indel | Breast | 1 |  |
| *BRCA1* | c.5406+5G>T | na | Expected Pathogenic | Splicing | Breast | 1 |  |
| *BRCA1* | c.5444G>A | p.Trp1815Ter | Pathogenic | Nonsense | Ovarian | 1 |  |
| *BRCA1* | c.5503C>T | p.Arg1835Ter | Pathogenic | Nonsense | Breast | 1 |  |
| *BRCA1* | c.68_69delAG | p.Glu23ValfsX16 | Pathogenic | Frameshift | Breast, Gastric, Ovarian, Ureter | 9 |  |
| *BRCA1* | c.815_824dup10 | p.Thr276AlafsX14 | Pathogenic | Frameshift | Breast | 1 |  |
| *BRCA1* | c.843_846delCTCA | p.Ser282TyrfsX15 | Pathogenic | Frameshift | Ovarian | 1 |  |
| *BRCA1* | c.869T>G | p.Leu290Ter | Pathogenic | Nonsense | None | 1 |  |
| *BRCA1* | c.895_896delGT | p.Val299ArgfsX4 | Pathogenic | Frameshift | Ovarian | 1 |  |
| *BRCA1* | Deletion exon 3 | na | Pathogenic | Gross deletion | Breast | 2 |  |
| *BRCA1* | Deletion exons 1-18 | na | Pathogenic | Gross deletion | Ovarian | 1 |  |
| *BRCA1* | Deletion exons 12-14 | na | Pathogenic | Gross deletion | Ovarian, Colon polyps | 1 |  |
| *BRCA1* | Deletion exons 1-22 | na | Pathogenic | Gross deletion | Breast | 1 |  |
| *BRCA1* | Deletion exons 12-21 | na | Pathogenic | Gross deletion | Breast | 1 |  |
| *BRCA1* | Deletion exons 14-16 | na | Pathogenic | Gross deletion | Fallopian tube | 1 |  |
| *BRCA1* | Deletion exons 7-12 | na | Pathogenic | Gross deletion | Ovarian | 1 |  |
| *BRCA1* | Deletion exons 7-23 | na | Pathogenic | Gross deletion | Breast | 1 |  |
| *BRCA1* | Deletion exons 7-8 | na | Pathogenic | Gross deletion | Breast | 1 |  |
| *BRCA1* | Duplication exon 12 | na | Pathogenic | Gross duplication | Breast, Carcinoid, Colon, Renal, Skin | 5 |  |
| *BRCA2* | c.1189_1190insTTAG | p.Gln397LeufsX25 | Pathogenic | Frameshift | Breast, Endometrial, Melanoma | 1 |  |
| *BRCA2* | c.1205delG | p.Gly402ValfsX2 | Pathogenic | Frameshift | None | 1 |  |
| *BRCA2* | c.1310_1313delAAGA | p.Lys437IlefsX22 | Pathogenic | Frameshift | Breast | 1 |  |
| *BRCA2* | c.1389_1390delAG | p.Val464GlyfsX3 | Pathogenic | Frameshift | Breast, Peritoneal | 1 |  |
| *BRCA2* | c.1411G>T | p.Glu471Ter | Pathogenic | Nonsense | Ovarian | 1 |  |
| *BRCA2* | c.145G>T | p.Glu49Ter | Pathogenic | Nonsense | None | 1 |  |
| *BRCA2* | c.1670T>G | p.Leu557Ter | Pathogenic | Nonsense | Breast | 1 |  |
| *BRCA2* | c.1754delA | p.Lys585ArgfsX29 | Pathogenic | Frameshift | Bile duct, Prostate | 1 |  |
| *BRCA2* | c.1755_1759delGAAAA | p.Lys585AsnfsX3 | Pathogenic | Frameshift | Breast, Fallopian tube, Pancreatic | 6 |  |
| *BRCA2* | c.1813dupA | p.Ile605AsnfsX11 | Pathogenic | Frameshift | Breast, Ovarian | 5 |  |
| *BRCA2* | c.1832C>A | p.Ser611Ter | Pathogenic | Nonsense | Breast, Thyroid | 1 |  |
| *BRCA2* | c.2092delC | p.Leu698TyrfsX32 | Pathogenic | Frameshift | Breast | 1 |  |
| *BRCA2* | c.2224C>T | p.Gln742Ter | Pathogenic | Nonsense | Breast | 1 |  |
| *BRCA2* | c.2426T>G | p.Leu809Ter | Pathogenic | Nonsense | Pancreatic | 1 |  |
| *BRCA2* | c.250C>T | p.Gln84Ter | Pathogenic | Nonsense | Breast | 1 |  |
| *BRCA2* | c.2692_2696delAGGAA | p.Arg898Ter | Pathogenic | Nonsense | Breast | 1 |  |
| *BRCA2* | c.2808_2811delACAA | p.Ala938ProfsX21 | Pathogenic | Frameshift | Breast | 1 |  |
| *BRCA2* | c.2830A>T | p.Lys944Ter | Pathogenic | Nonsense | Ovarian | 1 |  |
| *BRCA2* | c.3103G>T | p.Glu1035Ter | Pathogenic | Nonsense | Breast | 1 |  |
| *BRCA2* | c.3167_3170delAAAA | p.Gln1056ArgfsX3 | Pathogenic | Frameshift | Breast | 1 |  |
| *BRCA2* | c.3172A>T | p.Lys1058Ter | Pathogenic | Nonsense | Breast | 1 |  |
| *BRCA2* | c.3264dupT | p.Gln1089SerfsX10 | Pathogenic | Frameshift | Breast, Gastric, Ovarian | 4 |  |
| *BRCA2* | c.3599_3600delGT | p.Cys2000Ter | Pathogenic | Nonsense | None | 1 |  |
| *BRCA2* | c.3847_3848delGT | p.Val1283LysfsX2 | Pathogenic | Frameshift | Breast, Ovarian, Thyroid | 3 |  |
| *BRCA2* | c.3860delA | p.Asn1287IlefsX6 | Pathogenic | Frameshift | None | 1 |  |
| *BRCA2* | c.3860dupA | p.Asn1287LysfsX2 | Pathogenic | Frameshift | None | 1 |  |
| *BRCA2* | c.3922G>T | p.Glu1308Ter | Pathogenic | Nonsense | Breast | 2 |  |
| *BRCA2* | c.4000_4001delTT | p.Leu1334ArgfsX3 | Pathogenic | Frameshift | Breast | 1 |  |
| *BRCA2* | c.4163_4164delCTinsA | p.Thr1388AsnfsX22 | Pathogenic | Frameshift | Hamartoma of the Breast, lipoma | 1 |  |
| *BRCA2* | c.4168_4169delTT | p.Leu1390GlyfsX12 | Pathogenic | Frameshift | Breast | 1 |  |
| *BRCA2* | c.426-12_426-8delGTTTT | na | Expected Pathogenic | Splicing | Breast | 1 |  |
| *BRCA2* | c.4398_4402delACATT | p.Leu1466PhefsX2 | Pathogenic | Frameshift | Breast, Ovarian | 2 |  |
| *BRCA2* | c.4449delA | p.Asp1484ThrfsX2 | Pathogenic | Frameshift | None | 1 |  |
| *BRCA2* | c.4456_4459delGTTA | p.Val1486AsnfsX5 | Pathogenic | Frameshift | Breast | 1 |  |
| *BRCA2* | c.4478_4481delAAAG | p.Glu1493ValfsX10 | Pathogenic | Frameshift | None | 1 |  |
| *BRCA2* | c.4588A>T | p.Lys1530Ter | Pathogenic | Nonsense | Breast | 1 |  |
| *BRCA2* | c.4631delA | p.Asn1544ThrfsX24 | Pathogenic | Frameshift | Breast | 2 |  |
| *BRCA2* | c.4876_4877delAA | p.Asn1626SerfsX12 | Pathogenic | Frameshift | Breast, Ovarian, Thyroid | 2 |  |
| *BRCA2* | c.4936_4939delGAAA | p.Glu1646GlnfsX23 | Pathogenic | Frameshift | Ovarian | 1 |  |
| *BRCA2* | c.4965C>G | p.Tyr1655Ter | Pathogenic | Nonsense | Breast, Ovarian | 2 |  |
| *BRCA2* | c.5073dupA | p.Trp1692MetfsX3 | Pathogenic | Frameshift | Colon, Endometrial | 1 |  |
| *BRCA2* | c.5217_5220delTTTA | p.Tyr1739Ter | Pathogenic | Nonsense | Ovarian | 1 |  |
| *BRCA2* | c.5238dupT | p.Asn1747Ter | Pathogenic | Nonsense | Breast | 1 |  |
| *BRCA2* | c.5350_5351delAA | p.Asn1784HisfsX2 | Pathogenic | Frameshift | Ovarian | 2 |  |
| *BRCA2* | c.5576_5579delTTAA | p.Ile1859LysfsX3 | Pathogenic | Frameshift | Breast, Fallopian tube | 2 |  |
| *BRCA2* | c.5614A>T | p.Lys1872Ter | Pathogenic | Nonsense | None | 1 |  |
| *BRCA2* | c.5616_5620delAGTAA | p.Lys1872AsnfsX2 | Pathogenic | Frameshift | Breast | 1 |  |
| *BRCA2* | c.5621_5624delTTAA | p.Ile1874ArgfsX34 | Pathogenic | Frameshift | Breast | 1 |  |
| *BRCA2* | c.5682C>G | p.Tyr1894Ter | Pathogenic | Nonsense | Breast | 3 |  |
| *BRCA2* | c.574_575delAT | p.Met192ValfsX13 | Pathogenic | Frameshift | Breast | 1 |  |
| *BRCA2* | c.5799_5802delCCAA | p.Asn1933LysfsX29 | Pathogenic | Frameshift | Anal | 1 |  |
| *BRCA2* | c.5828delC | p.Ser1943LeufsX20 | Pathogenic | Frameshift | Prostate, Colon polyps | 1 |  |
| *BRCA2* | c.5851_5854delAGTT | p.Ser1951TrpfsX11 | Pathogenic | Frameshift | None | 1 |  |
| *BRCA2* | c.5946delT | p.Ser1982ArgfsX22 | Pathogenic | Frameshift | Breast, Colon, Pancreatic, Prostate, Polyposis | 5 |  |
| *BRCA2* | c.6068_6072delACCAG | p.Asp2023AlafsX24 | Pathogenic | Frameshift | Breast | 1 |  |
| *BRCA2* | c.6082_6086delGAAGA | p.Glu2028LysfsX19 | Pathogenic | Frameshift | Breast | 1 |  |
| *BRCA2* | c.6267_6269delGCAinsC | p.Glu2089AspfsX2 | Pathogenic | Frameshift | Breast | 1 |  |
| *BRCA2* | c.6275_6276delTT | p.Leu2092ProfsX7 | Pathogenic | Frameshift | Ovarian | 3 |  |
| *BRCA2* | c.631+2T>G | na | Pathogenic | Splicing | Hematologic | 1 |  |
| *BRCA2* | c.6373dupA | p.Thr2125AsnfsX4 | Pathogenic | Frameshift | Pancreatic | 1 |  |
| *BRCA2* | c.6405_6409delCTTAA | p.Asn2135LysfsX3 | Pathogenic | Frameshift | Breast | 1 |  |
| *BRCA2* | c.6444dupT | p.Ile2149TyrfsX2 | Pathogenic | Frameshift | Breast | 1 |  |
| *BRCA2* | c.6486_6489delACAA | p.Lys2162AsnfsX5 | Pathogenic | Frameshift | Breast, Ovarian | 3 |  |
| *BRCA2* | c.658_659delGT | p.Val220IlefsX4 | Pathogenic | Frameshift | Breast, Rectal, Colon polyps | 4 |  |
| *BRCA2* | c.670_673dupGATA | p.Thr225ArgfsX5 | Pathogenic | Frameshift | Sarcoma | 1 |  |
| *BRCA2* | c.6952C>T | p.Arg2318Ter | Pathogenic | Nonsense | Breast | 1 |  |
| *BRCA2* | c.7007G>A | na | Pathogenic | Splicing | Breast | 1 |  |
| *BRCA2* | c.7069_7070delCT | p.Leu2357ValfsX2 | Pathogenic | Frameshift | Breast | 1 |  |
| *BRCA2* | c.7379_7380insG | p.Asn2460LysfsX15 | Pathogenic | Frameshift | Breast | 1 |  |
| *BRCA2* | c.7588delC | p.Gln2530LysfsX21 | Pathogenic | Frameshift | Breast | 1 |  |
| *BRCA2* | c.7618-1G>A | na | Pathogenic | Splicing | Breast | 2 |  |
| *BRCA2* | c.771_775delTCAAA | p.Asn257LysfsX17 | Pathogenic | Frameshift | Breast | 1 |  |
| *BRCA2* | c.778_779delGA | p.Glu260SerfsX15 | Pathogenic | Frameshift | Endometrial | 1 |  |
| *BRCA2* | c.7976G>A | na | Pathogenic | Splicing | Breast, Melanoma, Skin | 1 |  |
| *BRCA2* | c.8009C>T | p.Ser2670Leu | Expected Pathogenic | Missense | Breast | 1 |  |
| *BRCA2* | c.8167G>C | p.Asp2723His | Pathogenic | Missense | Breast, Male Breast, Ovarian | 3 |  |
| *BRCA2* | c.8168A>C | p.Asp2723Ala | Expected Pathogenic | Missense | Breast | 1 |  |
| *BRCA2* | c.8174G>A | p.Trp2725Ter | Pathogenic | Nonsense | Breast | 1 |  |
| *BRCA2* | c.8297delC | p.Thr2766AsnfsX11 | Pathogenic | Frameshift | Breast | 1 |  |
| *BRCA2* | c.8331+1G>A | na | Pathogenic | Splicing | None | 1 |  |
| *BRCA2* | c.8394_8396delTAGinsAA | p.Arg2799AsnfsX22 | Pathogenic | Frameshift | Breast, Pancreatic | 1 |  |
| *BRCA2* | c.8487+1G>A | na | Pathogenic | Splicing | Breast, Sarcoma | 1 |  |
| *BRCA2* | c.891_899  delAACAGTTGT  insGATACTTCAG | p.Thr298IlefsX7 | Pathogenic | Frameshift | Breast | 1 |  |
| *BRCA2* | c.8978C>G | p.Ser2993Ter | Pathogenic | Nonsense | Breast | 1 |  |
| *BRCA2* | c.9097dupA | p.Thr3033AsnfsX11 | Pathogenic | Frameshift | Breast | 1 |  |
| *BRCA2* | c.9118-2A>G | na | Pathogenic | Splicing | Bladder | 1 |  |
| *BRCA2* | c.9253dupA | p.Thr3085AsnfsX26 | Pathogenic | Frameshift | Breast | 1 |  |
| *BRCA2* | c.9257-1G>C | na | Pathogenic | Splicing | Ovarian | 1 |  |
| *BRCA2* | c.9294C>G | p.Tyr3098Ter | Pathogenic | Nonsense | None | 1 |  |
| *BRCA2* | c.9331G>T | p.Glu3111Ter | Pathogenic | Nonsense | Breast, Ovarian | 1 |  |
| *BRCA2* | c.9648+1G>C | na | Pathogenic | Splicing | None | 1 |  |
| *BRCA2* | c.9728delC | p.Pro3243LeufsX6 | Pathogenic | Frameshift | None | 1 |  |
| *BRCA2* | Deletion exon 27 | na | Pathogenic | Gross deletion | Breast, Colon polyps | 1 |  |
| *BRIP1* | c.1066C>T | p.Arg356Ter | Pathogenic | Nonsense | Breast | 1 |  |
| *BRIP1* | c.1126_1127delCA | p.Gln376AsnfsX18 | Pathogenic | Frameshift | Breast | 1 |  |
| *BRIP1* | c.1156A>T | p.Lys386Ter | Pathogenic | Nonsense | Breast | 1 |  |
| *BRIP1* | c.1201_1204dupTGTG | p.Ala402ValfsX21 | Pathogenic | Frameshift | Ovarian, Skin | 1 |  |
| *BRIP1* | c.1315C>T | p.Arg439Ter | Pathogenic | Nonsense | Breast | 1 |  |
| *BRIP1* | c.1372G>T | p.Glu458Ter | Pathogenic | Nonsense | Breast, Glioblastoma, Colon polyps | 3 |  |
| *BRIP1* | c.139C>A | p.Pro47Thr | Expected Pathogenic | Missense | None | 1 |  |
| *BRIP1* | c.139C>G | p.Pro47Ala | Pathogenic | Missense | Breast, Hematologic, Ovarian | 9 |  |
| *BRIP1* | c.2038_2039dupTT | p.Leu680PhefsX9 | Pathogenic | Frameshift | Breast | 1 |  |
| *BRIP1* | c.2108delAinsTCC | p.Lys703IlefsX3 | Pathogenic | Frameshift | Ovarian | 1 |  |
| *BRIP1* | c.2255_2256delAA | p.Lys752ArgfsX12 | Pathogenic | Frameshift | Ovarian | 2 |  |
| *BRIP1* | c.2273dupT | p.Ala759SerfsX6 | Pathogenic | Frameshift | Endometrial | 1 |  |
| *BRIP1* | c.2392C>T | p.Arg798Ter | Pathogenic | Nonsense | Breast, Colorectal, Ovarian | 5 |  |
| *BRIP1* | c.2400C>G | p.Tyr800Ter | Pathogenic | Nonsense | Colon polyps | 1 |  |
| *BRIP1* | c.2492+2dupT | na | Expected Pathogenic | Splicing | None | 1 |  |
| *BRIP1* | c.627+1G>A | na | Pathogenic | Splicing | Ovarian | 1 |  |
| *CDH1* | c.1565+1G>A | na | Pathogenic | Splicing | Diffuse Gastric | 1 | No |
| *CDH1* | c.1979dupT | p.Asp662Ter | Pathogenic | Nonsense | None | 1 | Yes |
| *CDH1* | c.707C>A | p.Ser236Ter | Pathogenic | Nonsense | Gastric- NOS | 1 | Yes, if diffuse |
| *CDH1* | c.715G>A | na | Expected Pathogenic | Splicing | Breast | 1 | No |
| *CDKN2A* | c.301G>T | p.Gly101Trp | Pathogenic | Missense | Pancreatic | 2 |  |
| *CDKN2A* | c.-34G>T | na | Pathogenic | Regulatory | Melanoma | 2 |  |
| *CHEK2* | c.1039G>A | p.Asp347Asn | Expected Pathogenic | Missense | Breast | 1 |  |
| *CHEK2* | c.1100delC | p.Thr367MetfsX15 | Pathogenic | Frameshift | Astrocytoma, Brain, Breast, Colorectal, Endometrial, Hematologic, Male Breast, Ovarian, Neuroendocrine tumor, Prostate, Renal, Skin, Thyroid, Colon polyps | 78 |  |
| *CHEK2* | c.1254delT | p.Phe418LeufsX19 | Pathogenic | Frameshift | Breast | 1 |  |
| *CHEK2* | c.1263delT | p.Ser422ValfsX15 | Pathogenic | Frameshift | Bladder, Breast, Colon, Hematologic, Male Breast, Prostate, Urethral, Colon polyps | 6 |  |
| *CHEK2* | c.1283C>T | p.Ser428Phe | Pathogenic | Missense | Breast, Endometrial, Hematologic, Male Breast, Ovarian, Prostate, Thyroid | 9 |  |
| *CHEK2* | c.1356G>A | p.Trp452Ter | Pathogenic | Nonsense | None | 1 |  |
| *CHEK2* | c.1368dupA | p.Glu457ArgfsX33 | Pathogenic | Frameshift | Breast | 1 |  |
| *CHEK2* | c.1427C>T | p.Thr476Met | Pathogenic | Missense | Breast, Ovarian | 10 |  |
| *CHEK2* | c.1555C>T | p.Arg519Ter | Pathogenic | Nonsense | Breast, Colon | 2 |  |
| *CHEK2* | c.190G>A | p.Glu64Lys | Expected Pathogenic | Missense | Breast, Colon, Ovarian | 5 |  |
| *CHEK2* | c.277delT | p.Trp93GlyfsX17 | Pathogenic | Frameshift | Breast, Melanoma | 1 |  |
| *CHEK2* | c.319+2T>A | na | Pathogenic | Splicing | Breast | 1 |  |
| *CHEK2* | c.349A>G | p.Arg117Gly | Expected Pathogenic | Missense | Breast, Colon, Melanoma, Renal, Thyroid, Colon polyps | 14 |  |
| *CHEK2* | c.405delA | p.Lys135AsnfsX26 | Pathogenic | Frameshift | Breast, Cervical | 1 |  |
| *CHEK2* | c.433C>T | p.Arg145Trp | Pathogenic | Missense | Endometrial | 1 |  |
| *CHEK2* | c.444+1G>A | na | Pathogenic | Splicing | Breast, Colon, Melanoma, Ovarian, Thyroid | 3 |  |
| *CHEK2* | c.470T>C | p.Ile157Thr | Expected Pathogenic | Missense | Bladder, Breast, Colon, Endometrial, Male Breast, Melanoma, Ovarian, Pilocytic astrocytoma, Renal, Colon polyps | 42 |  |
| *CHEK2* | c.483_485delAGA | p.Glu161del | Expected Pathogenic | In-frame deletion | Ovarian | 1 |  |
| *CHEK2* | c.499G>A | p.Gly167Arg | Expected Pathogenic | Missense | Breast | 1 |  |
| *CHEK2* | c.507delT | p.Phe169LeufsX2 | Pathogenic | Frameshift | Bladder, Breast, Endometrial | 2 |  |
| *CHEK2* | c.524dupT | p.Gly176ArgfsX10 | Pathogenic | Frameshift | Appendix | 1 |  |
| *CHEK2* | c.565A>G | p.Ile189Val | Expected Pathogenic | Missense | Breast | 1 |  |
| *CHEK2* | c.591delA | p.Val198PhefsX7 | Pathogenic | Frameshift | Breast | 1 |  |
| *CHEK2* | c.793-1G>A | na | Pathogenic | Splicing | Breast | 1 |  |
| *CHEK2* | c.917G>C | p.Gly306Ala | Expected Pathogenic | Missense | Breast | 3 |  |
| *CHEK2* | Deletion exons 6-7 | na | Pathogenic | Gross deletion | Melanoma | 1 |  |
| *CHEK2* | Deletion exons 9-10 | na | Pathogenic | Gross deletion | Breast, Colon polyps | 3 |  |
| *EPCAM* | Deletion exons 8-9 | na | Pathogenic | Gross deletion | Colon | 1 |  |
| *EPCAM* | Deletion of Entire *EPCAM*  Gene and *MSH2* exons 1-7 | na | Pathogenic | Gross deletion | Colon | 1 |  |
| *FANCC* | c.1302dupT | p.Gly435TrpfsX83 | Pathogenic | Frameshift | None | 1 |  |
| *FANCC* | c.1387_1388delTC | p.Ala464ProfsX53 | Pathogenic | Frameshift | Breast | 1 |  |
| *FANCC* | c.1642C>T | p.Arg548Ter | Pathogenic | Nonsense | Breast, Colon polyps | 3 |  |
| *FANCC* | c.319C>T | p.Gln107Ter | Pathogenic | Nonsense | Breast | 1 |  |
| *FANCC* | c.355_360delTCTCATinsA | p.Ser119AsnfsX8 | Pathogenic | Frameshift | Breast | 3 |  |
| *FANCC* | c.37C>T | p.Gln13Ter | Pathogenic | Nonsense | Breast | 1 |  |
| *FANCC* | c.456+4A>T | na | Pathogenic | Splicing | Breast, Melanoma, Ovarian, Pancreatic | 7 |  |
| *FANCC* | c.487_490delGAGA | p.Glu163IlefsX30 | Pathogenic | Frameshift | Ovarian | 1 |  |
| *FANCC* | c.553C>T | p.Arg185Ter | Pathogenic | Nonsense | Breast, Ovarian | 3 |  |
| *FANCC* | c.67delG | p.Asp23IlefsX23 | Pathogenic | Frameshift | Breast, Endometrial | 2 |  |
| *FANCC* | c.-78-2A>G | na | Pathogenic | Splicing | Breast, Melanoma | 1 |  |
| *MLH1* | c.1039-1G>A | na | Pathogenic | Splicing | Colon, Sarcoma | 1 |  |
| *MLH1* | c.1050delA | p.Gly351AspfsX16 | Pathogenic | Frameshift | Colon, Sebaceous carcinoma | 1 |  |
| *MLH1* | c.1489dupC | p.Arg497ProfsX6 | Pathogenic | Frameshift | Colon, Endometrial | 2 |  |
| *MLH1* | c.155_156delAA | p.Lys52ArgfsX26 | Pathogenic | Frameshift | Colon | 1 |  |
| *MLH1* | c.1731G>A | na | Pathogenic | Splicing | Colon | 1 |  |
| *MLH1* | c.1852_1854delAAG | p.Lys618del | Pathogenic | In-frame deletion | Colon | 2 |  |
| *MLH1* | c.199G>A | p.Gly67Arg | Pathogenic | Missense | Colon, Colon polyp | 1 |  |
| *MLH1* | c.2065C>T | p.Gln689Ter | Pathogenic | Nonsense | Breast, Colon | 1 |  |
| *MLH1* | c.208-3C>G | na | Expected Pathogenic | Splicing | Colon | 1 |  |
| *MLH1* | c.209_215delAAGAAGA | p.Lys70IlefsX20 | Pathogenic | Frameshift | Colon | 1 |  |
| *MLH1* | c.2110dupG | p.Val704GlyfsX19 | Pathogenic | Frameshift | Colon | 1 |  |
| *MLH1* | c.306G>T | p.Glu102Asp | Expected Pathogenic | Missense | Colon, Colon polyps | 2 |  |
| *MLH1* | c.320T>G | p.Ile107Arg | Pathogenic | Missense | Colorectal | 1 |  |
| *MLH1* | c.350C>T | p.Thr117Met | Pathogenic | Missense | Colon | 1 |  |
| *MLH1* | c.380G>T | na | Expected Pathogenic | Splicing | Colorectal | 1 |  |
| *MLH1* | c.453+1G>T | na | Pathogenic | Splicing | Colon | 1 |  |
| *MLH1* | c.503dupA | p.Asn168LysfsX4 | Pathogenic | Frameshift | Colon | 1 |  |
| *MLH1* | c.589-2A>G | na | Pathogenic | Splicing | Colon, Endometrial, Leiomyosarcoma | 2 |  |
| *MLH1* | c.676C>T | p.Arg226Ter | Pathogenic | Nonsense | Breast, Colon | 2 |  |
| *MLH1* | c.677G>T | na | Expected Pathogenic | Splicing | Breast, Colon polyps | 1 |  |
| *MLH1* | c.67G>T | p.Glu23Ter | Pathogenic | Nonsense | Colon, Colon polyps | 1 |  |
| *MLH1* | c.791-2A>G | na | Pathogenic | Splicing | Colon | 1 |  |
| *MLH1* | c.971dupA | p.Arg325AlafsX37 | Pathogenic | Frameshift | Colon | 1 |  |
| *MLH1* | Deletion exon 16 | na | Pathogenic | Gross deletion | Colorectal | 1 |  |
| *MLH1* | Deletion exons 1-13 | na | Pathogenic | Gross deletion | Breast, Colon, Endometrial, Pancreatic, Colon polyps | 2 |  |
| *MLH1* | Deletion exons 1-6 | na | Pathogenic | Gross deletion | Colon | 1 |  |
| *MLH1* | Deletion exons 16-19 | na | Pathogenic | Gross deletion | Colon, Endometrial, Ovarian, Small bowel | 2 |  |
| *MLH1* | Deletion exons 2-3 | na | Pathogenic | Gross deletion | Endometrial | 1 |  |
| *MLH1* | Duplication exons 6-12 | na | Pathogenic | Gross duplication | Colon, Duodenal | 2 |  |
| *MSH2* | c.1157dupA | p.Asp386GlufsX3 | Pathogenic | Frameshift | Endometrial | 1 |  |
| *MSH2* | c.1165C>T | p.Arg389Ter | Pathogenic | Nonsense | Colon | 1 |  |
| *MSH2* | c.11dupA | p.Pro5AlafsX77 | Pathogenic | Frameshift | Colon | 1 |  |
| *MSH2* | c.1226_1227delAG | p.Gln409ArgfsX7 | Pathogenic | Frameshift | Endometrial | 1 |  |
| *MSH2* | c.1373T>G | p.Leu458Ter | Pathogenic | Nonsense | Breast | 1 |  |
| *MSH2* | c.1525A>T | p.Lys509Ter | Pathogenic | Nonsense | Ovarian, Endometrial | 1 |  |
| *MSH2* | c.1744delG | p.Val582SerfsX8 | Pathogenic | Frameshift | Rhabdomyosarcoma | 1 |  |
| *MSH2* | c.1786_1788delAAT | p.Asn596del | Pathogenic | In-frame deletion | Endometrial | 1 |  |
| *MSH2* | c.1906G>C | p.Ala636Pro | Pathogenic | Missense | Colon, Thyroid, Gastric polyps | 2 |  |
| *MSH2* | c.1916_1919delATGC | p.His639LeufsX45 | Pathogenic | Frameshift | Rectal | 1 |  |
| *MSH2* | c.2038C>T | p.Arg680Ter | Pathogenic | Nonsense | Colon, Sebaceous neoplasm | 2 |  |
| *MSH2* | c.2334C>A | p.Cys778Ter | Pathogenic | Nonsense | Bladder, Colon, Prostate | 1 |  |
| *MSH2* | c.2647delA | p.Ile883LeufsX9 | Pathogenic | Frameshift | Ovarian | 1 |  |
| *MSH2* | c.289C>T | p.Gln97Ter | Pathogenic | Nonsense | Appendiceal, Colon, Throat | 1 |  |
| *MSH2* | c.571_573delCTC | p.Leu191del | Expected Pathogenic | In-frame deletion | Colon | 1 |  |
| *MSH2* | c.686_687delAA | p.Lys229SerfsX2 | Pathogenic | Frameshift | Colon | 1 |  |
| *MSH2* | c.70C>T | p.Gln24Ter | Pathogenic | Nonsense | Endometrial, Peritoneal | 1 |  |
| *MSH2* | c.859G>T | p.Gly287Ter | Pathogenic | Nonsense | None | 1 |  |
| *MSH2* | c.932delA | p.Asn311ThrfsX20 | Pathogenic | Frameshift | Colon polyps | 1 |  |
| *MSH2* | c.942+3A>T | na | Pathogenic | Splicing | Bladder, Colorectal, Ovarian, Sebaceous gland, Sebaceous adenoma | 8 |  |
| *MSH2* | c.998G>A | p.Cys333Tyr | Pathogenic | Missense | Breast, Ovarian | 1 |  |
| *MSH2* | Deletion exon 8 | na | Pathogenic | Gross deletion | Ampulla of vater, Colon polyps | 1 |  |
| *MSH2* | Deletion exons 1-2 | na | Pathogenic | Gross deletion | None | 1 |  |
| *MSH2* | Deletion exons 1-6 | na | Pathogenic | Gross deletion | Breast, Colon, Endometrial, Ovarian | 5 |  |
| *MSH2* | Deletion exons 3-8 | na | Pathogenic | Gross deletion | Colon, Endometrial | 2 |  |
| *MSH2* | Deletion exons 8-15 | na | Pathogenic | Gross deletion | None | 1 |  |
| *MSH6* | c.1135_1139delAGAGA | p.Arg379Ter | Pathogenic | Nonsense | Cervical | 1 |  |
| *MSH6* | c.1190_1191delAT | p.Tyr397CysfsX3 | Pathogenic | Frameshift | Endometrial, Colon polyps | 1 |  |
| *MSH6* | c.1241G>A | p.Trp414Ter | Pathogenic | Nonsense | Endometrial, Borderline ovarian tumor | 1 |  |
| *MSH6* | c.1634_1635delAA | p.Lys545ArgfsX17 | Pathogenic | Frameshift | Colorectal | 1 |  |
| *MSH6* | c.1634_1637delAAGA | p.Lys545ArgfsX25 | Pathogenic | Frameshift | Breast, Endometrial | 2 |  |
| *MSH6* | c.1805C>G | p.Ser602Ter | Pathogenic | Nonsense | Renal, Sarcoma, Primitive neuroectodermal tumor | 1 |  |
| *MSH6* | c.2057G>A | p.Gly686Asp | Expected Pathogenic | Missense | Breast | 1 |  |
| *MSH6* | c.2194C>T | p.Arg732Ter | Pathogenic | Nonsense | Endometrial | 1 |  |
| *MSH6* | c.2731C>T | p.Arg911Ter | Pathogenic | Nonsense | Small bowel | 1 |  |
| *MSH6* | c.2832_2833delAA | p.Ile944MetfsX4 | Pathogenic | Frameshift | Endometrial, Colon polyps | 1 |  |
| *MSH6* | c.3142C>T | p.Gln1048Ter | Pathogenic | Nonsense | None | 1 |  |
| *MSH6* | c.3155_3156delAG | p.Glu1052ValfsX13 | Pathogenic | Frameshift | Endometrial | 2 |  |
| *MSH6* | c.3202C>T | p.Arg1068Ter | Pathogenic | Nonsense | Endometrial | 1 |  |
| *MSH6* | c.3261delC | p.Phe1088SerfsX2 | Pathogenic | Frameshift | Breast. Colon, Endometrial, Ovarian, Angiomyolipomas, Colon polyps | 4 |  |
| *MSH6* | c.3261dupC | p.Phe1088LeufsX5 | Pathogenic | Frameshift | Breast, Colon, Endometrial, Renal, Colon polyps | 3 |  |
| *MSH6* | c.3487G>T | p.Glu1163Ter | Pathogenic | Nonsense | Ovarian | 1 |  |
| *MSH6* | c.3523_3524dupAC | p.Arg1176LeufsX9 | Pathogenic | Frameshift | Colon | 1 |  |
| *MSH6* | c.3690delA | p.Val1231LeufsX9 | Pathogenic | Frameshift | Endometrial | 1 |  |
| *MSH6* | c.3746_3749dupACCA | p.His1250GlnfsX26 | Pathogenic | Frameshift | Endometrial | 1 |  |
| *MSH6* | c.3934_3937dupGTTA | p.Ile1313SerfsX7 | Pathogenic | Frameshift | Breast, Colorectal, Endometrial | 1 |  |
| *MSH6* | c.3939_3940dupTC | p.Gln1314LeufsX14 | Pathogenic | Frameshift | Bladder, Colorectal | 1 |  |
| *MSH6* | c.3939_3957dup19 | p.Ala1320SerfsX5 | Pathogenic | Frameshift | Endometrial, Ovarian | 1 |  |
| *MSH6* | c.3984_3487dupGTCA | p.Leu1330AlafsX12 | Pathogenic | Frameshift | Endometrial | 1 |  |
| *MSH6* | c.3991C>T | p.Arg1331Ter | Pathogenic | Nonsense | Breast, Colon | 1 |  |
| *MSH6* | c.4001G>A | p.Arg1334Gln | Pathogenic | Missense | Breast, Colorectal | 1 |  |
| *MSH6* | c.468_471delAAAG | p.Glu158ProfsX15 | Pathogenic | Frameshift | Endometrial, Ovarian | 1 |  |
| *MSH6* | c.602_603delAG | p.Glu201AlafsX17 | Pathogenic | Frameshift | Breast, Lobular Breast | 3 |  |
| *MSH6* | c.702_703insT | p.Thr235TyrfsX5 | Pathogenic | Frameshift | Colon, Colon polyps | 1 |  |
| *MSH6* | c.892C>T | p.Arg298Ter | Pathogenic | Nonsense | Breast, Endometrial | 1 |  |
| *MUTYH* | c.1145G>A | p.Gly382Asp | Pathogenic | Missense | Bladder, Breast, Endometrial | 1 |  |
| *MUTYH* | c.1185_1186dupGG | p.Glu396GlyfsX43 | Pathogenic | Frameshift | Bladder, Breast, Endometrial | 1 |  |
| *MUTYH* | c.1187G>A | p.Gly396Asp | Pathogenic | Missense | Bladder, Colorectal, Testicular, Colorectal polyps, Polyposis, Sebaceous adenomas | 8 |  |
| *MUTYH* | c.1227_1228dupGG | p.Glu410GlyfsX43 | Pathogenic | Frameshift | Colon polyps, Sebaceous adenomas | 1 |  |
| *MUTYH* | c.391T>A | p.Trp131Arg | Pathogenic | Missense | Colon, Colon polyps | 1 |  |
| *MUTYH* | c.536A>G | p.Tyr179Cys | Pathogenic | Missense | Bladder, Colon, Endometrial, Pancreatic, Testicular, Colon polyps, Polyposis | 3 |  |
| *MUTYH* | c.545G>A | p.Arg182His | Pathogenic | Missense | Colon, Endometrial, Pancreatic | 1 |  |
| *NBN* | c.105_135del31 | p.Ile35MetfsX4 | Pathogenic | Frameshift | Breast, Glioblastoma | 1 |  |
| *NBN* | c.1397+2T>A | na | Pathogenic | Splicing | Breast | 1 |  |
| *NBN* | c.2117C>G | p.Ser706Ter | Pathogenic | Nonsense | Breast | 1 |  |
| *NBN* | c.2140C>T | p.Arg714Ter | Pathogenic | Nonsense | None | 1 |  |
| *NBN* | c.657_661delACAAA | p.Lys219AsnfsX16 | Pathogenic | Frameshift | Breast, Lobular Breast, Ovarian | 6 |  |
| *NBN* | c.698_701delAACA | p.Lys233SerfsX5 | Pathogenic | Frameshift | Breast, Lung | 2 |  |
| *NBN* | c.817dupA | p.Thr273AsnfsX12 | Pathogenic | Frameshift | Breast, Colon polyps, Trichilemmomas | 1 |  |
| *NBN* | Deletion exons 15-16 | na | Expected Pathogenic | Gross deletion | Breast | 1 |  |
| *PALB2* | c.1059delA | p.Lys353AsnfsX3 | Pathogenic | Frameshift | Breast | 1 |  |
| *PALB2* | c.109-2A>G | na | Pathogenic | Splicing | None | 1 |  |
| *PALB2* | c.1240C>T | p.Arg414Ter | Pathogenic | Nonsense | Breast, Colon, Male Breast, Thyroid, Colon polyps | 3 |  |
| *PALB2* | c.1317delG | p.Phe440LeufsX12 | Pathogenic | Frameshift | Breast | 1 |  |
| *PALB2* | c.1592delT | p.Leu531CysfsX30 | Pathogenic | Frameshift | Breast, Ovarian | 2 |  |
| *PALB2* | c.1675_1676delCAinsTG | p.Gln559Ter | Pathogenic | Nonsense | Breast | 1 |  |
| *PALB2* | c.172_175delTTGT | p.Gln60ArgfsX7 | Pathogenic | Frameshift | Breast, Ovarian, Pancreatic | 3 |  |
| *PALB2* | c.1924delA | p.Met642CysfsX18 | Pathogenic | Frameshift | Breast | 1 |  |
| *PALB2* | c.2006delA | p.Glu669GlyfsX3 | Pathogenic | Frameshift | Breast, Colon, Prostate | 2 |  |
| *PALB2* | c.2052delC | p.Arg686GlyfsX23 | Pathogenic | Frameshift | Lobular Breast | 1 |  |
| *PALB2* | c.2120delC | p.Pro707LeufsX2 | Pathogenic | Frameshift | Breast, Thyroid | 1 |  |
| *PALB2* | c.212-2A>G | na | Pathogenic | Splicing | Breast | 1 |  |
| *PALB2* | c.2154delG | p.Arg718SerfsX14 | Pathogenic | Frameshift | Breast | 1 |  |
| *PALB2* | c.2229T>A | p.Tyr743Ter | Pathogenic | Nonsense | Breast | 1 |  |
| *PALB2* | c.226delA | p.Ile76TyrfsX101 | Pathogenic | Frameshift | None | 1 |  |
| *PALB2* | c.2390_2396delAACCTAC | p.Gln797ProfsX52 | Pathogenic | Frameshift | Breast | 2 |  |
| *PALB2* | c.2559C>T | na | Expected Pathogenic | Splicing | Pancreatic | 1 |  |
| *PALB2* | c.2642_2645dupGTTG | p.Cys882TrpfsX3 | Pathogenic | Frameshift | Breast | 2 |  |
| *PALB2* | c.2727_2728delTT | p.Thr911LeufsX16 | Pathogenic | Frameshift | Breast | 1 |  |
| *PALB2* | c.2834+1G>A | na | Pathogenic | Splicing | Breast | 1 |  |
| *PALB2* | c.2920_2921delAA | p.Lys974GlufsX5 | Pathogenic | Frameshift | Breast | 1 |  |
| *PALB2* | c.3026delC | p.Pro1009LeufsX6 | Pathogenic | Frameshift | Breast | 1 |  |
| *PALB2* | c.3113G>A | p.Trp1038Ter | Pathogenic | Nonsense | Breast, Colon polyps | 6 |  |
| *PALB2* | c.3202-1G>C | na | Pathogenic | Splicing | Breast | 1 |  |
| *PALB2* | c.3256C>T | p.Arg1086Ter | Pathogenic | Nonsense | Adrenal gland, Breast | 2 |  |
| *PALB2* | c.3456dupA | p.Pro1153ThrfsX4 | Pathogenic | Frameshift | Breast | 3 |  |
| *PALB2* | c.3549C>A | p.Tyr1183Ter | Pathogenic | Nonsense | Breast | 4 |  |
| *PALB2* | c.3549C>G | p.Tyr1183Ter | Pathogenic | Nonsense | Breast, Ovarian, Pancreatic | 3 |  |
| *PALB2* | c.688G>T | p.Glu230Ter | Pathogenic | Nonsense | Breast | 1 |  |
| *PALB2* | c.757_758delCT | p.Leu253IlefsX3 | Pathogenic | Frameshift | Breast, Endometrial | 1 |  |
| *PALB2* | c.758dupT | p.Ser254IlefsX3 | Pathogenic | Frameshift | Breast | 1 |  |
| *PALB2* | c.948delC | p.Thr317GlnfsX5 | Pathogenic | Frameshift | Breast | 1 |  |
| *PALB2* | Deletion exon 11 | na | Pathogenic | Gross deletion | Breast, Cervical | 3 |  |
| *PALB2* | Deletion exon 7 | na | Pathogenic | Gross deletion | Breast | 1 |  |
| *PMS2* | c.1112_1113delATinsTTTA | p.Asn371IlefsX2 | Pathogenic | Frameshift | Breast | 1 |  |
| *PMS2* | c.1239delA | p.Asp414ThrfsX34 | Pathogenic | Frameshift | Endometrial | 1 |  |
| *PMS2* | c.1376C>A | p.Ser459Ter | Pathogenic | Nonsense | Colorectal | 1 |  |
| *PMS2* | c.137G>T | p.Ser46Ile | Pathogenic | Missense | Cervical, Gastric, Melanoma, Colon polyps | 4 |  |
| *PMS2* | c.1579_1580delAG | p.Arg527GlyfsX14 | Pathogenic | Frameshift | Breast | 1 |  |
| *PMS2* | c.1831dupA | p.Ile611AsnfsX2 | Pathogenic | Frameshift | Colon, Glioblastoma | 1 |  |
| *PMS2* | c.1A>G | p.Met1? | Pathogenic | Loss of initiation codon | Breast | 1 |  |
| *PMS2* | c.2113G>A | p.Glu705Lys | Pathogenic | Missense | Breast, Colon, Colon polyps | 2 |  |
| *PMS2* | c.2117delA | p.Lys706SerfsX19 | Pathogenic | Frameshift | Ovarian | 1 |  |
| *PMS2* | c.2174+1G>A | na | Pathogenic | Splicing | Colon | 1 |  |
| *PMS2* | c.2T>A | p.Met1? | Pathogenic | Loss of initiation codon | Colon | 1 |  |
| *PMS2* | c.2T>C | p.Met1? | Pathogenic | Loss of initiation codon | Breast | 1 |  |
| *PMS2* | c.400C>T | p.Arg134Ter | Pathogenic | Nonsense | Ovarian | 1 |  |
| *PMS2* | c.697C>T | p.Gln233Ter | Pathogenic | Nonsense | Colon polyps | 1 |  |
| *PMS2* | c.736_741  delCCCCCT  insTGTGTGTGAAG | p.Pro246CysfsX3 | Pathogenic | Frameshift | Colon, Glioblastoma, Ovarian | 2 |  |
| *PMS2* | c.823C>T | p.Gln275Ter | Pathogenic | Nonsense | Endometrial | 1 |  |
| *PMS2* | c.943C>T | p.Arg315Ter | Pathogenic | Nonsense | Breast | 2 |  |
| *PMS2* | c.989-1G>T | na | Pathogenic | Splicing | Colon polyps | 1 |  |
| *PMS2* | Deletion exon 10 | na | Pathogenic | Gross deletion | None | 1 |  |
| *PMS2* | Deletion exon 8 | na | Pathogenic | Gross deletion | Endometrial | 1 |  |
| *PMS2* | Deletion exons 5-9 | na | Pathogenic | Gross deletion | Breast, Colon | 1 |  |
| *PMS2* | Deletion exons 6-8 | na | Pathogenic | Gross deletion | Cervical, Colon | 1 |  |
| *PTEN* | c.112C>T | p.Pro38Ser | Expected Pathogenic | Missense | Breast, Colon | 1 |  |
| *PTEN* | c.165-1G>A | na | Pathogenic | Splicing | Colon polyps | 1 |  |
| *PTEN* | c.253+1G>T | na | Pathogenic | Splicing | Breast | 1 |  |
| *PTEN* | c.254-2A>G | na | Pathogenic | Splicing | Colon Polyps, Gastric Polyps | 1 |  |
| *PTEN* | c.289C>T | p.Gln97Ter | Pathogenic | Nonsense | None | 1 |  |
| *PTEN* | c.367C>T | p.His123Tyr | Pathogenic | Missense | Breast, Hematologic | 1 |  |
| *PTEN* | c.437T>A | p.Leu146Ter | Pathogenic | Nonsense | Breast | 1 |  |
| *PTEN* | c.547_549delAAGinsT | p.Lys183Ter | Pathogenic | Nonsense | Breast | 1 |  |
| *PTEN* | c.737C>T | p.Pro246Leu | Pathogenic | Missense | Colon | 1 |  |
| *PTEN* | c.955dupA | p.Thr319AsnfsX6 | Pathogenic | Frameshift | Colon polyps, Lipoma | 1 |  |
| *PTEN* | Deletion exons 6-8 | na | Pathogenic | Gross deletion | Colon polyps | 1 |  |
| *RAD51C* | c.1026+5_1026+7delGTA | na | Expected Pathogenic | Splicing | Ovarian | 1 |  |
| *RAD51C* | c.224dupA | p.Tyr75Ter | Pathogenic | Nonsense | Breast | 1 |  |
| *RAD51C* | c.394dupA | p.Thr132AsnfsX23 | Pathogenic | Frameshift | Breast | 1 |  |
| *RAD51C* | c.404+2T>C | na | Pathogenic | Splicing | Ovarian | 1 |  |
| *RAD51C* | c.502A>T | p.Arg168Ter | Pathogenic | Nonsense | Breast | 1 |  |
| *RAD51C* | c.577C>T | p.Arg193Ter | Pathogenic | Nonsense | Ovarian | 1 |  |
| *RAD51C* | c.706-2A>G | na | Pathogenic | Splicing | Breast, Fallopian tube, Melanoma | 4 |  |
| *RAD51C* | c.837+4_837+7delAGTA | na | Expected Pathogenic | Splicing | Breast | 1 |  |
| *RAD51C* | c.905-3_906delCAGGG | na | Pathogenic | Splicing | Breast | 1 |  |
| *RAD51C* | c.93delG | p.Phe32SerfsX8 | Pathogenic | Frameshift | Hematologic, Ovarian | 2 |  |
| *RAD51C* | c.965+1G>A | na | Pathogenic | Splicing | Breast | 1 |  |
| *RAD51C* | c.97C>T | p.Gln33Ter | Pathogenic | Nonsense | Endometrial | 1 |  |
| *RAD51C* | Deletion exon 5 | na | Pathogenic | Gross deletion | None | 1 |  |
| *RAD51D* | c.1A>G | p.Met1? | Pathogenic | Loss of initiation codon | Ovarian, Colon polyps | 1 |  |
| *RAD51D* | c.270_271dupTA | p.Lys91IlefsX13 | Pathogenic | Frameshift | Breast | 2 |  |
| *RAD51D* | c.326dupC | p.Gly110ArgfsX2 | Pathogenic | Frameshift | Colorectal | 1 |  |
| *RAD51D* | c.363delA | p.Ala122GlnfsX14 | Pathogenic | Frameshift | Breast | 1 |  |
| *RAD51D* | c.694C>T | p.Arg232Ter | Pathogenic | Nonsense | Breast | 1 |  |
| *RAD51D* | c.748delC | p.His250ThrfsX2 | Pathogenic | Frameshift | Breast, Ovarian | 4 |  |
| *RAD51D* | Deletion exon 10 | na | Expected Pathogenic | Gross deletion | Breast | 1 |  |
| *RAD51D* | Deletion exon 3 | na | Pathogenic | Gross deletion | Ovarian | 1 |  |
| *RAD51D* | Deletion exons 1-10 | na | Expected Pathogenic | Gross deletion | Breast, Melanoma | 1 |  |
| *SMAD4* | c.1239C>A | p.Tyr413Ter | Pathogenic | Nonsense | Duodenal, Polyposis | 1 |  |
| *SMAD4* | c.1245_1248delCAGA | p.Asp415GlufsX20 | Pathogenic | Frameshift | Colon polyps | 1 |  |
| *SMAD4* | c.1351_1375del25 | p.Ala451LeufsX17 | Pathogenic | Frameshift | Colon polyps | 1 |  |
| *STK11* | Deletion exon 9-10 | na | Pathogenic | Gross deletion | Colon, Colon polyps | 1 |  |
| *STK11* | Deletion exons 4-9 | na | Pathogenic | Gross deletion | Colorectal, Colon polyps | 1 |  |
| *STK11* | Deletion of 5'UTR | na | Pathogenic | Gross deletion | Gastric | 1 |  |
| *TP53* | c.1101-2A>G | na | Pathogenic | Splicing | Breast | 1 | No |
| *TP53* | c.1125delG | p.Gln375HisfsX47 | Pathogenic | Frameshift | Breast, Sarcoma | 1 | Yes |
| *TP53* | c.314G>A | p.Gly105Asp | Pathogenic | Missense | Breast, Hematologic | 1 | Yes |
| *TP53* | c.328delC | p.Arg110ValfsX13 | Pathogenic | Frameshift | Breast | 1 | Yes |
| *TP53* | c.365_366delTG | p.Val122AspfsX26 | Pathogenic | Frameshift | None | 1 | Yes |
| *TP53* | c.481G>A | p.Ala161Thr | Expected Pathogenic | Missense | Breast | 1 | Yes |
| *TP53* | c.493C>T | p.Gln165Ter | Pathogenic | Nonsense | Breast | 1 | Yes |
| *TP53* | c.580C>T | p.Leu194Phe | Pathogenic | Missense | Breast | 1 | Yes |
| *TP53* | c.637C>T | p.Arg213Ter | Pathogenic | Nonsense | Breast | 1 | No |
| *TP53* | c.709A>G | p.Met237Val | Pathogenic | Missense | Breast | 1 | Yes |
| *TP53* | c.733G>T | p.Gly245Cys | Pathogenic | Missense | Ovarian | 1 | No |
| *TP53* | c.742C>T | p.Arg248Trp | Pathogenic | Missense | Breast, Lung | 1 | Yes |
| *TP53* | c.743G>A | p.Arg248Gln | Pathogenic | Missense | Breast | 1 | Yes |
| *TP53* | c.817C>T | p.Arg273Cys | Pathogenic | Missense | Colon polyps | 1 | No |
| *TP53* | c.818G>A | p.Arg273His | Pathogenic | Missense | Breast, Sarcoma | 1 | No |
| *TP53* | c.848G>A | p.Arg283His | Expected Pathogenic | Missense | Breast | 1 | No |
| *TP53* | Deletion exon 11 | na | Pathogenic | Gross deletion | Breast | 1 | Yes |
| *TP53* | Deletion of entire *TP53* gene | na | Pathogenic | Gross deletion | Ovarian | 1 | Yes |
| *VHL* | c.154G>T | p.Glu52Ter | Pathogenic | Nonsense | Breast | 1 |  |
| *VHL* | c.445G>T | p.Ala149Ser | Pathogenic | Missense | Melanoma, Pancreatic | 1 |  |
| *XRCC2* | c.545delA | p.Lys182SerfsX3 | Pathogenic | Frameshift | Breast | 1 |  |
| *XRCC2* | c.96delT | p.Phe32LeufsX30 | Pathogenic | Frameshift | Breast | 2 |  |

na, not applicable

1. The following transcripts were used in analysis: *APC* NM_000038.5; *ATM* NM_000051.3; *AXIN2* NM_004655.3; *BARD1* NM_000465.2; *BMPR1A* NM_004329.2; *BRCA1* NM_007294.3; *BRCA2* NM_000059.3; *BRIP1* NM_032043.2; *CDH1* NM_004360.3; *CDK4* NM_000075.3; *CDKN2A* NM_000077.4(p16), NM_058195.3(p14-ARF); *CHEK2* NM_007194.3; *EPCAM* NM_002354.2; *FANCC* NM_000136.2; *MLH1* NM_000249.3; *MSH2* NM_000251.2; *MSH6* NM_000179.2; *MUTYH* NM_001128425.1; *NBN* NM_002485.4; *PALB2* NM_024675.3; *PMS2* NM_000535.5; *PTEN* NM_000314.4; *RAD51C* NM_058216.1; *RAD51D* NM_002878.3; *SMAD4* NM_005359.5; *STK11* NM_000455.4; *TP53* NM_000546.5; *VHL* NM_000551.3; *XRCC2* NM_005431.1
2. Gross deletions/duplications include copy number variation at the level of one or more exons.
3. Clinical histories include all cancers, and other findings such as polyps, reported by all individuals with a particular variant. If not otherwise indicated, the diagnosis listed is a malignant neoplasm.
4. For *CDH1* and *TP53*, it is noted whether patients met clinical testing criteria for *CDH1*^25^ or Li-Fraumeni syndrome (LFS) , respectively. Patients with *TP53* variants were categorized as fulfilling LFS criteria if they met classic LFS,^20^ LFS-like,^21^ 2009 Chompret,^22,23^ or NCCN guideline criteria for LFS/*TP53* testing.^24^
