# Supplementary material for: Pathogenic and likely pathogenic variant prevalence among the first 10,000 patients referred for next-generation cancer panel testing
Source: Genet Med. 2015 Dec 17;18(8):823–32. doi: 10.1038/gim.2015.166 (PMC4985612; doi:10.1038/gim.2015.166)
Supplement: Supplementary Table S2 [file gim2015166x2.doc]

**Table S2. Pathogenic and Likely Pathogenic Variants by Gene and Variant Type.**

Susswein L, Marshall M, Nusbaum R *et al.* Pathogenic and likely pathogenic variant prevalence among the first 10,000 patients referred for next-generation cancer panel testing. *Genetics in Medicine.* 2015

**Pathogenic (P) and Likely Pathogenic (LP) Variants**

**P+LP (LP only, if identified)**

| **Gene** | **Premature termination** | **Gross**  **deletion/**  **duplication** | **Splicing** | **Loss of initiation codon** | **Missense** | **Regulatory** | **In-frame** | **Gene Total** | **% of all**  **Positive Variants** |
| --- | --- | --- | --- | --- | --- | --- | --- | --- | --- |
| ***APC*** | 18 | 1 | 2 | - | - |  | - | **21** | **2.2%** |
| ***ATM*** | 49 | 4 | 19(1) | - | 14(5) |  | 4 | **90(6)** | **9.6%** |
| ***AXIN2*** | - | - | - | 1 | - |  | - | **1** | **0.1%** |
| ***BARD1*** | 9 | - | - | 1 | - |  | - | **10** | **1.1%** |
| ***BMPR1A*** | 2 | 1 | - | - | - |  | - | **3** | **0.3%** |
| ***BRCA1*** | 87 | 15 | 12(2) | - | 10(1) |  | 1 | **125(3)** | **13.3%** |
| ***BRCA2*** | 113 | 1 | 11(1) | - | 5(2) |  | - | **130(3)** | **13.9%** |
| ***BRIP1*** | 19 | - | 2(1) | - | 10(1) |  | - | **31(2)** | **3.3%** |
| ***CDH1*** | 2 | - | 2(1) | - | - |  | - | **4(1)** | **0.4%** |
| ***CDKN2A*** | 2 | - | - | - | 2 | 2 | - | **4** | **0.4%** |
| ***CHEK2*** | 95 | 4 | 5 | - | 87(67) |  | 1(1) | **192(68)** | **20.5%** |
| ***EPCAM*** | - | 2 | - | - | - |  | - | **2** | **0.2%** |
| ***FANCC*** | 16 | - | 8 | - | - |  | - | **24** | **2.6%** |
| ***MLH1*** | 12 | 9 | 9(3) | - | 5(2) |  | 2 | **37(5)** | **3.9%** |
| ***MSH2*** | 17 | 10 | 8 | - | 3 |  | 2(1) | **40(1)** | **4.3%** |
| ***MSH6*** | 36 | - | - | - | 2(1) |  | - | **38(1)** | **4.1%** |
| ***MUTYH*** | 2 | - | - | - | 14 |  | - | **16** | **1.7%** |
| ***NBN*** | 12 | 1(1) | 1 | - | - |  | - | **14(1)** | **1.5%** |
| ***PALB2*** | 48 | 4 | 5(1) | - | - |  | - | **57(1)** | **6.1%** |
| ***PMS2*** | 13 | 4 | 2 | 3 | 6 |  | - | **28** | **3.0%** |
| ***PTEN*** | 4 | 1 | 3 | - | 3(1) |  | - | **11(1)** | **1.2%** |
| ***RAD51C*** | 7 | 1 | 9(2) | - | - |  | - | **17(2)** | **1.8%** |
| ***RAD51D*** | 9 | 3(2) | - | 1 | - |  | - | **13(2)** | **1.4%** |
| ***SMAD4*** | 3 | - | - | - | - |  | - | **3** | **0.3%** |
| ***STK11*** | - | 3 | - | - | - |  | - | **3** | **0.3%** |
| ***TP53*** | 5 | 2 | 1 | - | 10(2) |  | - | **18(2)** | **1.9%** |
| ***VHL*** | 1 | - | - | - | 1 |  | - | **2** | **0.2%** |
| ***XRCC2*** | 3 | - | - | - | - |  | - | **3** | **0.3%** |
| **Total** | 582 | 66(3) | 99(12) | 6 | 172(82) | 2 | 10(2) | **937 (99)** |  |
| ***%*** | **62.1%** | **7.0%** | **10.6%** | **0.6%** | **18.4%** | **0.2%** | **1.1%** |  |  |

Premature termination variants include frameshift and nonsense, due to either single nucleotide substitutions or small insertions/deletions. Gross deletions/duplications include copy number variation at the level of one or more exons.
