# Supplementary material for: Pathogenic and likely pathogenic variant prevalence among the first 10,000 patients referred for next-generation cancer panel testing
Source: Genet Med. 2015 Dec 17;18(8):823–32. doi: 10.1038/gim.2015.166 (PMC4985612; doi:10.1038/gim.2015.166)
Supplement: Supplementary Table S3 [file gim2015166x3.doc]

| **Two High Risk Genes** | | |  |  |  |  |
| --- | --- | --- | --- | --- | --- | --- |
| **Case** | **Gene** | **cDNA** | **Protein** | **Classification** | **Sex** | **Personal history of cancer** |
|  |  |  |  |  |  |  |
| 1 | ***PMS2*** | c.1831dupA | p.Ile611AsnfsX2 | Pathogenic | M | Glioblastoma, Colon |
|  | ***PMS2*** | c.736_741delCCCCCT | p.Pro246CysfsX3 | Pathogenic |  |  |
|  |  | insTGTGTGTGAAG |  |  |  |  |
|  |  |  |  |  |  |  |
| 2 | ***BMPR1A*** | c.262G>T | p.Glu88Ter | Pathogenic | M | Unaffected |
|  | ***TP53*** | c.817C>T | p.Arg273Cys | Pathogenic |  |  |
|  |  |  |  |  |  |  |
| 3 | ***BRCA1*** | c.68_69delAG | p.Glu23ValfsX17 | Pathogenic | F | Gastric |
|  | ***PMS2*** | c.137G>T | p.Ser46Ile | Pathogenic |  |  |
|  |  |  |  |  |  |  |
| 4 | ***BRCA2*** | c.1670T>G | p.Leu557Ter | Pathogenic | F | Breast |
|  | ***VHL*** | c.154G>T | p.Glu52Ter | Pathogenic |  |  |
|  |  |  |  |  |  |  |
| 5 | ***BRCA2*** | c.3264dupT | p.Gln1089SerfsX10 | Pathogenic | F | Gastric |
|  | ***STK11*** | Deletion of 5'UTR | na | Pathogenic |  |  |
|  |  |  |  |  |  |  |
|  |  |  |  |  |  |  |
| **One High Risk Gene, One Moderate/Unknown Risk** | | | | | | |
| **Case** | **Gene** | **cDNA** | **Protein** | **Classification** | **Sex** | **Personal history of cancer** |
| 6 | ***MUTYH*** | c.1185_1186dupGG | p.Glu396GlyfsX43 | Pathogenic | F | Uterine, Breast, Bladder |
|  | ***MUTYH*** | c.1145G>A | p.Gly382Asp | Pathogenic |  |  |
|  | ***CHEK2*** | c.507delT | p.Phe169LeufsX2 | Pathogenic |  |  |
|  |  |  |  |  |  |  |
| 7 | ***BRCA2*** | c.2092delC | p.Leu698TyrfsX32 | Pathogenic | F | Breast |
|  | ***FANCC*** | c.355_360 | p.Ser119AsnfsX8 | Pathogenic |  |  |
|  |  | delTCTCATinsA |  |  |  |  |
|  |  |  |  |  |  |  |
| 8 | ***BRCA2*** | c.4588A>T | p.Lys1530Ter | Pathogenic | F | Breast |
|  | ***NBN*** | c.698_701 | p.Lys233SerfsX5 | Pathogenic |  |  |
|  |  | delAACA |  |  |  |  |
|  |  |  |  |  |  |  |
| 9 | ***BRCA2*** | c.891_899 | p.Thr298IlefsX7 | Pathogenic | F | Bilateral Breast |
|  |  | delAACAGTTGT |  |  |  |  |
|  |  | insGATACTTCAG |  |  |  |  |
|  | ***CHEK2*** | c.349A>G | p.Arg117Gly | Likely Pathogenic | |  |
|  |  |  |  |  |  |  |
| 10 | ***BRCA1*** | c.3748G>T | p.Glu1250Ter | Pathogenic | F | Breast, Colon |
|  | ***CHEK2*** | c.470T>C | p.Ile157Thr | Likely Pathogenic | |  |
|  |  |  |  |  |  |  |
| 11 | ***PALB2*** | c.172_175delTTGT | p.Gln60ArgfsX7 | Pathogenic | F | Ovarian |
|  | ***PMS2*** | c.400C>T | p.Arg134ter | Pathogenic |  |  |
|  |  |  |  |  |  |  |
| 12 | ***BRCA1*** | c.3456dupA | p.Pro1153ThrfsX4 | Pathogenic | F | Breast |
|  | ***PALB2*** | c.3481_3491del11 | p.Glu1161PhefsX3 | Pathogenic |  |  |
|  |  |  |  |  |  |  |
| 13 | ***BRCA1*** | c.1175_1214del40 | p.Leu392GlnfsX5 | Pathogenic | M | Male Breast, Prostate, Urethral, |
|  | ***CHEK2*** | c.1263delT | p.Ser422ValfsX15 | Pathogenic |  | Bladder, Colon, Lymphoma, Colon Polyps |
|  |  |  |  |  |  |  |
| 14 | ***BARD1*** | c.1690C>T | p.Gln564Ter | Pathogenic | F | Ovarian |
|  | ***MSH6*** | c.3487G>T | p.Glu1163Ter | Pathogenic |  |  |
|  |  |  |  |  |  |  |
| 15 | ***APC*** | na | Deletion of Entire APC Gene | Pathogenic | F | Hematologic |
|  | ***ATM*** | c.3526delC | p.Leu1176CysfsX5 | Pathogenic |  |  |
|  |  |  |  |  |  |  |
| 16 | ***CHEK2*** | c.470T>C | p.Ile157Thr | Likely Pathogenic | F | Breast |
|  | ***MSH6*** | c.2057G>A | p.Gly686Asp | Likely Pathogenic | |  |
|  |  |  |  |  |  |  |
| 17 | ***ATM*** | c.6976-2A>C | IVS47-2 | Pathogenic | M | Colon |
|  | ***BRCA2*** | c.5946delT | p.Ser1982ArgfsX22 | Pathogenic |  |  |
|  |  |  |  |  |  |  |
| **Two Genes with Moderate/Unknown Risk** | | | | | | |
| **Case** | **Gene** | **cDNA** | **Protein** | **Classification** | **Sex** | **Personal history of cancer** |
|  |  |  |  |  |  |  |
| 18 | ***ATM*** | c.640delT | p.Ser214ProfsX16 | Pathogenic | F | Unaffected |
|  | ***CHEK2*** | c.470T>C | p.Ile157Thr | Likely Pathogenic | |  |
|  |  |  |  |  |  |  |
|  |  |  |  |  |  |  |
| 19 | ***ATM*** | c.8266A>T | p.Lys2756Ter | Pathogenic | F | Bilateral Breast |
|  | ***CHEK2*** | c.1263delT | p.Ser422ValfsX15 | Pathogenic |  |  |
|  |  |  |  |  |  |  |
| 20 | ***FANCC*** | c.487_490delGAGA | p.Glu163IlefsX30 | Pathogenic | F | Ovarian |
|  | ***RAD51C*** | c.404+2T>C | IVS2+2 | Pathogenic |  |  |
|  |  |  |  |  |  |  |
| 21 | ***ATM*** | c.2897_2899 | p.Val966GlyfsX6 | Pathogenic | F | Breast |
|  |  | delTTCinsGCCAA |  |  |  |  |
|  | ***CHEK2*** | c.917G>C | p.Gly306Ala | Likely Pathogenic | |  |
|  |  |  |  |  |  |  |
| 22 | ***ATM*** | c.549_550delTA | p.His183GlnfsX6 | Pathogenic | M | Colon |
|  | ***ATM*** | c.7913G>A | p.Trp2638Ter | Pathogenic |  |  |
|  |  |  |  |  |  |  |
| 23 | ***ATM*** | c.3245_3247 | p.His1082LeufsX14 | Pathogenic | F | Brain |
|  |  | delATCinsTGAT |  |  |  |  |
|  | ***CHEK2*** | c.470T>C | p.Ile157Thr | Likely Pathogenic | |  |
|  |  |  |  |  |  |  |
| 24 | ***ATM*** | c.8266A>T | p.Lys2756Ter | Pathogenic | F | Breast |
|  | ***CHEK2*** | c.1100delC | p.Thr367MetfsX15 | Pathogenic |  |  |
|  |  |  |  |  |  |  |
| 25 | ***CHEK2*** | c.1100delC | p.Thr367MetfsX15 | Pathogenic | F | Neuroendocrine tumor |
|  | ***CHEK2*** | c.1100delC | p.Thr367MetfsX15 | Pathogenic |  |  |
|  |  |  |  |  |  |  |
| 26 | ***CHEK2*** | c.1100delC | p.Thr367MetfsX15 | Pathogenic | F | Unaffected |
|  | ***CHEK2*** | c.1100delC | p.Thr367MetfsX15 | Pathogenic |  |  |
|  |  |  |  |  |  |  |
| 27 | ***CHEK2*** | c.349A>G | p.Arg117Gly | Likely Pathogenic | F | Breast |
|  | ***CHEK2*** | c.1100delC | p.Thr367MetfsX15 | Pathogenic |  |  |
|  |  |  |  |  |  |  |
| 28 | ***CHEK2*** | c.470T>C | p.Ile157Thr | Likely Pathogenic | F | Unaffected |
|  | ***CHEK2*** | c.1100delC | p.Thr367MetfsX15 | Pathogenic |  |  |
|  |  |  |  |  |  |  |
